# Single‐Molecule Mechanoresistivity by Intermetallic Bonding

**DOI:** 10.1002/anie.202418062

**Published:** 2024-11-06

**Authors:** Amit Sil, Chiara E. Spano, Yahia Chelli, Simon J. Higgins, Sara Sangtarash, Gianluca Piccinini, Mariagrazia Graziano, Richard J. Nichols, Hatef Sadeghi, Andrea Vezzoli

**Affiliations:** ^1^ Department of Chemistry University of Liverpool Crown Street Liverpool L69 7ZD UK; ^2^ Department of Electronics and Telecommunications Politecnico di Torino Corso Duca degli Abruzzi 10129 Torino Italy; ^3^ Istituto Nazionale di Ricerca Metrologica (INRiM) Strada Delle Cacce 91 10135 Torino Italy; ^4^ Device Modelling Group School of Engineering University of Warwick Coventry CV4 7AL United Kingdom; ^5^ Department of Applied Science and Technology Politecnico di Torino Corso Duca degli Abruzzi 10129 Torino Italy

**Keywords:** Mechanoresistive Molecular Junctions, Molecular Electronics, Pt(II) Molecular Wires, Metal-Molecule Interfaces, Ion-Metal Interactions

## Abstract

The metal‐electrode interface is key to unlocking emergent behaviour in all organic electrified systems, from battery technology to molecular electronics. In the latter, interfacial engineering has enabled efficient transport, higher device stability, and novel functionality. Mechanoresistivity – the change in electrical behaviour in response to a mechanical stimulus and a pathway to extremely sensitive force sensors – is amongst the most studied phenomena in molecular electronics, and the molecule‐electrode interface plays a pivotal role in its emergence, reproducibility, and magnitude. In this contribution, we show that organometallic molecular wires incorporating a Pt(II) cation show mechanoresistive behaviour of exceptional magnitude, with conductance modulations of more than three orders of magnitude upon compression by as little as 1 nm. We synthesised series of cyclometalated Pt(II) molecular wires, and used scanning tunnelling microscopy – break junction techniques to characterise their electromechanical behaviour. Mechanoresistivity arises from an interaction between the Pt(II) cation and the Au electrode triggered by mechanical compression of the single‐molecule device, and theoretical modelling confirms this hypothesis. Our study provides a new tool for the design of functional molecular wires by exploiting previously unreported ion‐metal interactions in single‐molecule devices, and develops a new framework for the development of mechanoresistive molecular junctions.

## Graphical abstract

A novel method to impart mechanoresistive behaviour to molecular junctions is described in this contribution, relying on metal‐ion interactions between Au electrodes and the Pt(II) centre of cyclometalated molecular wires. Junctions can be cycled between the low‐conductance and high‐conductance state by mechanical compression and relaxation, with changes in transport efficiency exceeding a factor of 10^3^.X/Twitter Handles:

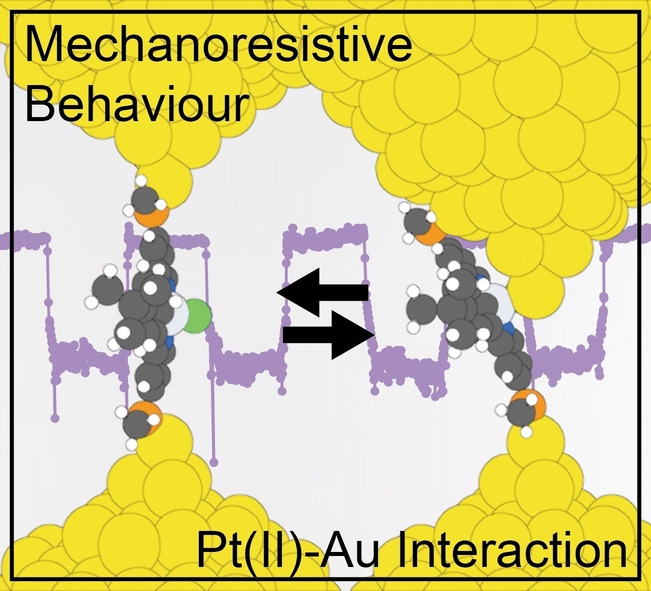

## Introduction

**Figure 1:**
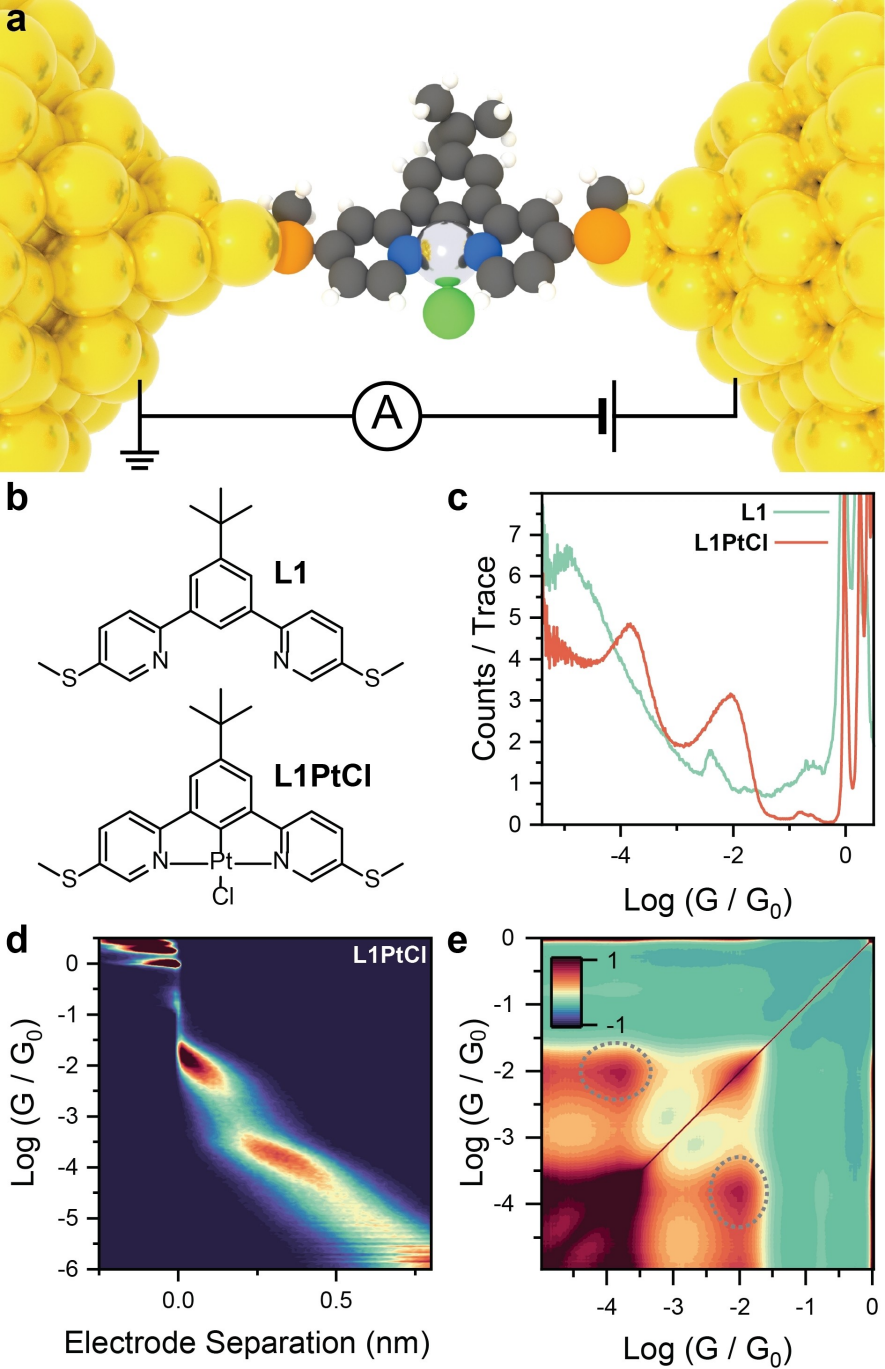
(a) Depiction of a single‐molecule junction incorporating a cyclometalated Pt(II) complex. (b) Structures of compounds L1 and L1PtCl. (c) STMBJ conductance histogram for L1 and L1PtCl. (d) 2D density map (conductance *vs* electrode separation) for L1PtCl. (e) Autocorrelogram for L1PtCl. The off‐axis features indicating correlation between the high and low conductance features are highlighted. All data acquired at 300 mV bias, with a 100μM concentration of the target compound in mesitylene. Plots in (c)‐(e) compiled from 4279 STMBJ traces for L1 and 5878 STMBJ traces for L1PtCl, using 100 bins per conductance decade and 100 bins per nm, with a sampling frequency of 20kHz . Colours in (a): C = grey, H = white, Pt = light grey, Au = yellow, S = orange, Cl = green, N = blue.

Single‐molecule junctions are nanoscale electronic devices made by a molecule “chemically soldered” to two metallic (or semiconducting) nanoelectrodes (Figure 1a). The structure of the molecule and its interface with the electrode can be engineered to impart the desired functionality, and atomic precision is granted by the bottom‐up approach to fabrication: molecules are synthesised with a well‐defined structure, and self‐assembly phenomena are exploited to obtain junctions with the desired structure. The effect of introducing metal centres in the molecular wire – as organometallic compounds or coordination complexes – has been investigated since the inception of molecular electronics.^[1]^ The nature of the metal centre strongly influences the ability to transport charge by changing the alignment of its frontier orbital to the Fermi energy of the electrodes,[^2^, ^3^] even allowing efficient long‐range transport.^[4]^ The ancillary ligand shell, comprising the ligands on the metallic centre not interfacing with the electrodes, can be further engineered to fine‐tune transport properties.[^5^, ^6^] The redox properties of organometallic, coordination and cluster compounds also allow the fabrication of devices that behave as efficient switches, triggered by an electrochemical potential[^7^, ^8^] or the electric field resulting from the source‐drain bias of the junction.[^9^, ^10^]

An attractive way to tune single‐molecule conductance is to exploit mechanoresistive phenomena^[11]^ (*e.g*. changes in conductance as response to mechanical stimuli), which are highly sought‐after in single‐entity devices as they could pave the way to the next generation of accurate pressure, force and displacement sensors. Molecular nanoelectromechanical systems^[11]^ are indeed an attractive alternative to the current approaches that rely on challenging top‐down nanofabrication of solid‐state cantilevers. Mechanical sensitivity has been imparted to molecular junctions either by exploiting force‐ or displacement‐induced changes in the metal‐molecule interface[^12^, ^13^, ^14^, ^15^, ^16^] or structural/electronic reorganisation of the molecular wire upon junction compression or stretching.[^17^, ^18^, ^19^, ^20^, ^21^] In molecular junctions incorporating organometallic fragments or coordinated metal centres, notable examples include mechanically‐triggered spin‐crossover, achieved in a bis(terpyridyl) Fe(II) complex^[22]^ and mechanically‐controlled quantum interference in ferrocene derivatives.^[23]^ Both of these are forms of internal electronic reorganisation of the molecule upon mechanical manipulation, and interfacial effects induced by the presence of a metallic centre have yet to be reported.

Intrigued by this opportunity, we decided to exploit the known interaction of Pt(II) salts with Au surfaces, both as single‐crystal^[24]^ and as polycrystalline^[25]^ substrates, to develop a novel way to impart mechanoresistivity to molecular junctions. In what follows, we demonstrate that the Pt(II) centre of a cyclometalated organoplatinum compound acts as an efficient interface to the Au electrodes, and its integration at the centre of a molecular wire terminated with other aurophilic functional groups enables reproducible and robust mechanical conductance switching of unprecedented magnitude.

## Results and Discussion

We started our investigation by synthesising the N C N ligand L1 and its Pt(II) complex L1PtCl (Figure 1b). The ligand L1 was synthesised by Suzuki‐Miyaura borylation of 1,3‐dibromo‐5‐(*tert*‐butyl)benzene, followed by Suzuki cross‐coupling with 2‐bromo(5‐methylthio)pyridine. Treatment of L1 with potassium tetrachloroplatinate in acetic acid gave L1PtCl. Synthetic routes and characterisation of the compounds used in this study can be found in the SI. We then used the Scanning Tunnelling Microscope Break Junction (STMBJ) technique^[26]^ to fabricate single‐molecule junctions and measure their charge transport characteristics. In this technique, a STM Au tip is driven into a Au substrate by a piezoelectric transducer to fabricate a microcontact having conductance $G\gg G_{0}$ , where $G_{0}$ is the quantum of conductance $\frac{2e^{2}}{h}≅77.48\;\mu S$ . The tip is then withdrawn at constant speed ($10\;nm\;s^{-1}$ in this study), causing the contact to thin down until, eventually, only an atomic contact having $G=G_{0}$ is left. Further withdrawal ruptures the atomic contact, leaving a pair of atomically sharp nanoelectrodes separated by a small distance due to snapback phenomena.^[27]^ Molecules provided with appropriate aurophilic termini can bridge the freshly formed nanogap, self‐assembling into a single‐molecule junction. Withdrawal of the tip is continued to stretch the junction to its most extended conformation, until its rupture. The tip is then driven again into the substrate to fabricate a fresh microcontact, and the whole process is repeated thousands of times to acquire statistically significant data. Current I through the junction is continuously acquired under a DC bias (V=300-600mV in this study) using a transimpedance amplifier, and conductance is calculated using Ohm's law as G=I/V . Data are then plotted as histograms and heatmaps, showing the distribution of conductance values (as $G/G_{0}$ ) and its relationship with the junction size. A brief description of the equipment used in this study and of the data acquisition, processing and analysis protocols can be found in the SI, while extensive technical details are given in our previous publication on the subject.[^7^, ^17^, ^28^]

STMBJ measurements on L1 and L1PtCl immediately showed the effect of complexation on charge‐transport properties (Figure 1c). The free ligand L1 gave a single pronounced peak at low conductance values ($∼10^{-5}{\;G}_{0}$ , close to the noise level of our instrumentation), not surprising since destructive quantum interference phenomena are expected due to the *meta* connectivity^[29]^ at the central phenyl ring. A small contribution can be observed at $∼10^{-2.4}\;G_{0}$ , which we attribute to transport through the pyridyl N (*e.g*. the junction being connected S‐N to the Au electrodes). On the other hand, the Pt(II) complex L1PtCl returned a well‐defined set of two peaks, centred approximately at $10^{-2.1}\;G_{0}$ and at $10^{-3.9}\;G_{0}$ . Analysis of the plateau lengths for L1 returned a value of 1.27±0.21nm (upon consideration of a snapback distance of 0.5 nm), in good agreement with the theoretical length calculated by density functional theory (DFT; see SI for details). In the case of L1PtCl, instead, the low conductance feature has length (1.08±0.10nm ) well commensurate with the theoretical S‐S distance, but the limited extension of the high‐conductance plateaux (0.70±0.06nm ) can only be rationalised if the organometallic Pt(II) centre is considered (Figure 1d). The DFT calculations, in fact, returned a S‐Pt distance of 0.68nm , in excellent agreement with our experimental values. We therefore postulated that, upon junction formation and at small electrode‐electrode separation, the molecule interacts with one electrode using one of the two thiomethyl termini, but at the other electrode the interaction is a Pt‐Au intermetallic bond, facilitated by the empty Pt(II) orbitals and the square planar structure of the cyclometalated complex. As discussed in the introduction, the interaction has been described in the literature, with evidence of well‐defined self‐assembled monolayers of Pt(II) species on Au substrates.^[24]^ As the junction is stretched, the Pt‐Au interaction is weakened and L1PtCl is then able to rearrange its interface to the electrodes to deliver the most extended junction configuration with a thiomethyl terminus interacting with each electrode. To test the robustness of our hypothesis, we performed autocorrelation analysis^[30]^ on our dataset, a technique originally developed for atomic point contacts^[31]^ that enables a visualisation of the correlation of different features in the conductance histogram, in a way similar to 2D techniques used in magnetic resonance spectroscopy.^[32]^ In brief, each conductance trace is logarithmically binned and the correlation coefficient between the counts in each bin is evaluated, accumulated, normalised for the entire dataset, and plotted as a 2D correlogram. When two conductance features are correlated, positive off‐axis features appear in the correlogram. Further details on the algorithm used is available in our previous publication on the subject.^[20]^ According to our model, the high conductance and low conductance features in the histogram should be highly correlated, as junctions would preferentially form in the compressed S‐Pt configuration at low electrode separation, eventually stretching to their most extended S‐S state during the STMBJ measurement. The correlogram in Figure 1e indeed shows strong off‐axis positive correlation between the high and low conductance features, thereby suggesting that both are consistently present in the single STMBJ traces as part of the junction evolution as the electrode separation is increased. Furthermore, this suggests the junctions are chemically robust and no decomposition of the Pt(II) species L1PtCl is occurring in the junction. If that were the case, we would not observe positive off‐axis features in the correlogram, as a decomposed L1PtCl would then not yield well‐resolved low‐conductance plateaux upon junction stretching.

**Figure 2:**
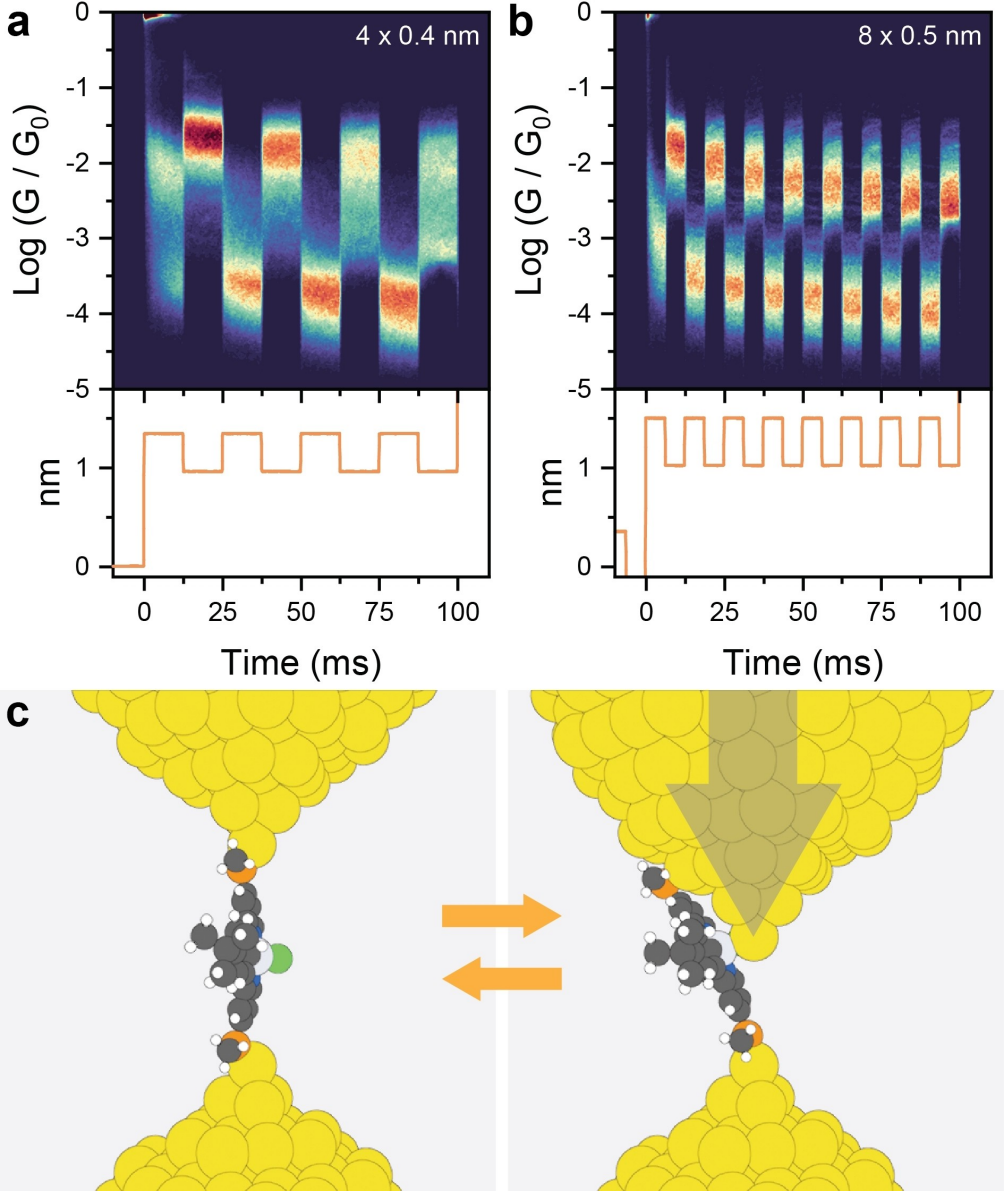
(a) Piezo‐modulation density map for L1PtCl using 4 x 0.4 nm amplitude. (b) Piezo‐modulation density map for L1PtCl using 8 x 0.5 nm amplitude. (c) Proposed mechanism of mechanical switching, with L1PtCl sliding along the electrode to expose the Pt(II) centre to the electrode as the junction is compressed. Plots in (a) and (b) compiled from 2820 and 3626 traces, respectively, using 100 bins per conductance decade and 2000 bins per second. The voltage signal applied to the piezoelectric transducer is reproduced below the density maps in (a) and (b). All data acquired with L1PtCl in 100μM concentration in mesitylene at 300mV bias. Colours in (c): C = grey, H = white, Pt = light grey, Au = yellow, S = orange, Cl = green, N = blue.

Since our data so far pointed us towards significant mechanoresistive phenomena in place in the junctions of L1PtCl, we performed piezo‐modulation experiments to verify their reliability and resilience. In these experiments, once the junction is in place in its extended configuration (*i.e*. after point contact rupture and self‐assembly of the target molecule) the tip withdrawal is paused, and a small modulation is applied to the voltage of the piezoelectric actuator. We started by applying 4×0.4nm modulation to our junctions, which delivered mechanoresistive behaviour, but as can be observed in Figure 2a, the reproducibility is far from ideal. Junctions fabricated with L1PtCl seem to require some initial training and in four modulations they fail to settle on stable values. This phenomenon has also been observed in permethyloligogermane molecular wires,^[33]^ and it was attributed to a modulation‐induced reshaping of the electrode structure and of the electrode‐molecule interfacial geometry towards the energy minima of both the high‐ and low‐conductance configurations. Therefore, we decided to apply a larger modulation amplitude (0.5nm ) at a higher frequency (8 modulations cycles in 100 ms) to promote faster mechanical annealing^[34]^ of the electrodes. These results are reported in Figure 2b, and the increased modulation amplitude and the additional modulation cycles allowed us to achieve full and reproducible switching between stable and consistent high and low conductance states once the junction has been sufficiently “trained”, with significant switching magnitude ($\frac{G_{HIGH}}{G_{LOW}}≅52$ ).

**Figure 3:**
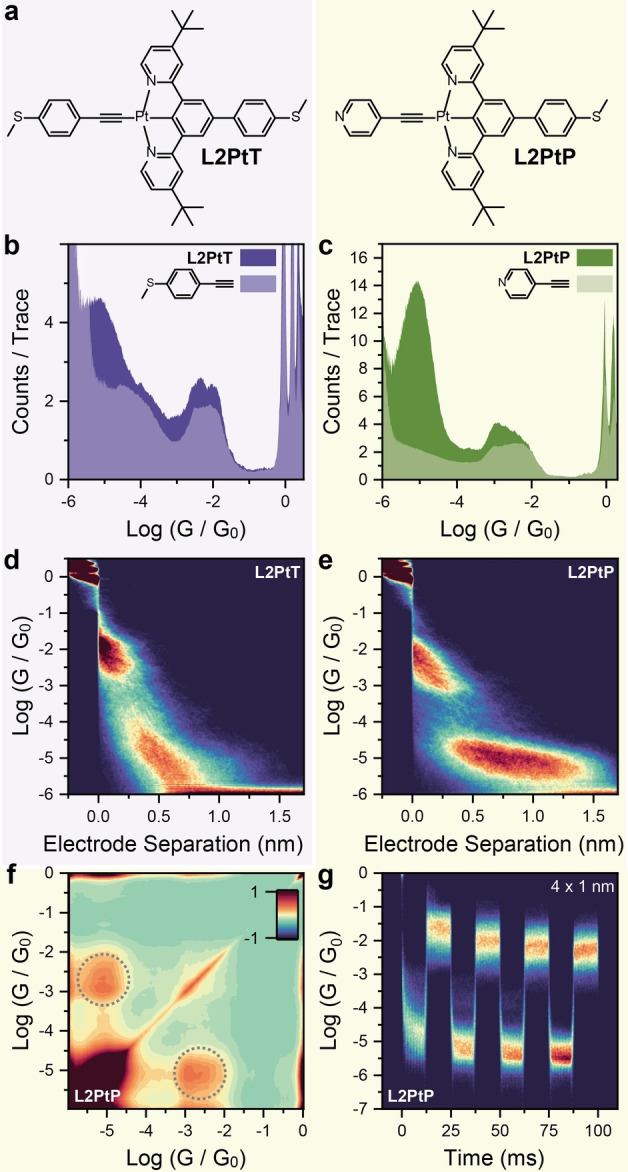
(a) structures of L2PtT and L2PtP. (b) Conductance histogram for L2PtT and its alkynyl fragment 4‐ethynylthioanisole. (c) Conductance histogram for L2PtP and its alkynyl fragment 4‐ethynylpyridine. (d) 2D density map for L2PtT, and (e) 2D density map for L2PtP. (f) Autocorrelogram for L2PtP. (g) Piezo‐modulation density map for L2PtP. (b) and (d) were compiled from 5002 traces, (c), (e), and (f) using 5792 traces and (g) using 2262 traces. All experiments on L2PtT and L2PtP performed at 600mV bias to reduce the noise floor of our instrument. Experiments on 4‐ethynylthioanisole and 4‐ethynylpyridine performed at 300mV bias. All experiments performed with a 100μM concentration of the target compound in mesitylene. Plots compiled with 100 bins per conductance decade, 100 bins per nanometre and 2000 bins per second.

Other phenomena could be in place, beyond the Au‐Pt(II) interaction (Figure 2c) we postulated being responsible for the observed mechanoresistive behaviour. A simple interpretation could be based on the formation of π ‐stacked dimers contributing to the low conductance feature. We can discount this explanation on the basis of (i) the presence of a *t*‐butyl substituent on L1PtCl that efficiently prevent stacking interaction with its steric bulk, and (ii) the analysis of the 2D conductance – electrode separation map, showing final junction break‐off well correlated with what is expected of a single‐molecule bridging the electrode‐electrode gap, and no features at the larger electrode separation generally found in π ‐stacked dimers.[^35^, ^36^] On the other hand, interactions between the Au electrode and the chloride ion cannot be as easily discounted, as halides have been shown to be possible aurophilic groups for the fabrication of molecular junctions.[^29^, ^37^] Similarly, interactions of the electrodes with the nitrogen atoms of the pyridyl rings, with a Pt‐N $\vec{\leftarrow }$ Au‐N reversible coordination switching rather than interactions with the Pt(II) centre, could be responsible for the observed mechanoresistivity. In order to provide a counter‐argument to these hypotheses and verify our proposed mechanism, we synthesised the compounds L2PtT and L2PtP (Figure 3a), which keep the N C N pincer structure with a cyclometalated Pt(II) centre, but the chloride ion is replaced by an alkynyl moiety, delivering an asymmetric structure. The synthetic process started with 1,3,5‐tribromobenzene, which was coupled to 4‐(thiomethyl)phenylboronic acid under Suzuki conditions, subjected to Suzuki‐Miyaura borylation to yield the diboronic ester and finally coupled to two equivalents of 4‐(*tert‐*butyl)‐2‐chloropyridine to give the free ligand L2. Treatment with potassium tetrachloroplatinate gave the Pt(II) complex L2PtCl which was converted to our target compounds L2PtT and L2PtP by reacting it with the corresponding terminal acetylide. Synthetic routes, procedures and characterisation can be found in the SI.

We performed STMBJ measurements on these compounds and on their various precursors. As expected, both L2PtT and L2PtP showed binary conductance contributions in the histogram (Figure 3b‐c), and analysis of the break‐off lengths (see SI) and 2D density maps (Figure 3d‐e) shows that in both cases the high conductance feature is again associated with a shorter break‐off length, while the less conductive feature extends to larger electrode separation. Interestingly, it proved rather difficult to stretch L2PtT to its full length, and we attribute this phenomenon to the destabilisation of the Au‐S interface due to the presence of the strongly electron‐deficient cyclometalated Pt(II) moiety. Junctions fabricated with L2PtP, on the other hand, could be extended to lengths commensurate with that of the molecular wire (theoretical values determined by DFT, see SI for details) owing to the stronger Au‐N interface. Comparison of the conductance histograms of L2PtT and L2PtP with those of 4‐ethynylthioanisole and 4‐ethynylpyridine (exploiting the aurophilic capabilities of terminal alkynes) shows that the high‐conductance feature arises from transport through that fragment only, with the 3’,5’‐dipyridyldiphenyl side not participating in transport in the high‐conductance configuration.

The high selectivity towards a single junction configuration in the compressed state of both L2PtT and L2PtP is indeed surprising, but can be rationalised through the stabilising interaction of the large 3,5’‐dipyridinylbenzene π ‐system with the Au electrode effectively creating a thermodynamic minimum. Further analysis on L2PtP demonstrates again the two contributions on the conductance histogram are well‐correlated, with strong off‐axis signatures in the correlogram (Figure 3f), suggesting that a similar mechanism of mechanoresistivity to that operating in L1PtCl is also in place here, and that the complex is stable under the experimental conditions. We then performed piezo‐modulation experiments on L2PtP (Figure 3g), with amplitude of 1 nm. Compounds L2PtP showed excellent mechanoresistive behaviour, with very little junction training needed to achieve stable and reproducible switching, and a magnitude $\frac{G_{HIGH}}{G_{LOW}}≅2700$ , at the time of writing and to our best knowledge the highest ever reported for mechanoresistive junctions. Compound L2PtP therefore acts as proof of the proposed switching mechanism. We can discount interactions with halides being responsible for the mechanoresistivity of L1PtCl as there is no halide present in L2PtP. We can also discount reversible Pt‐N $\vec{\leftarrow }$ Au‐N coordination switching at the cyclometalated core, as this would return different conductance values than those observed: the excellent match of the high‐conductance signature with that of the corresponding 4‐ethynylpyridine rules out any alternative explanation for mechanoresistivity and highlights the excellent electronic transparency of the Pt(II)‐Au interface, akin to a fully‐open quantum channel. Importantly, the excellent reversibility that can be observed in Figure 3g over multiple modulation cycles confirms the stability of the molecular wire, with no possibility of transmetallation of the terminal alkyne from the Pt(II) centre to the Au electrode.

In order to understand the nature of the Au‐Pt(II) interaction, we then measured the conductance of analogues of L2PtP lacking one of the aurophilic termini: in L2PtCl the 4‐ethynylpyridine end is replaced by a chloride ion, while in L3PtP the thioanisolyl end has been completely removed. Details on the synthesis, characterisation, and single‐molecule charge transport measurements of these control experiments can be found in the SI. In both cases, a clear STMBJ signal that could be attributed to transport from one aurophilic end to the Pt(II) centre could not be recorded. In the case of L2PtCl, while a peak is visible in the conductance histogram, the 2D density map shows very short break‐off (∼0.3nm ) not commensurate with the Pt‐S distance even accounting for electrode snapback, and poor charge transport efficiency ($G\;≅10^{-4.7}G_{0}$ ). In the case of L3PtP no clear peak or feature in the 2D density map was visible. We attribute these results to the inability of the Pt(II)‐Au interface alone to support robust and resilient junction formation. Therefore, the Pt(II) centre and the stronger aurophilic moiety (*i.e*. the thioanisolyl or pyridyl moiety) behave together like a hemilabile^[13]^ ligand: conductance is low in the *hemi* stage where only the strong S‐Au or N‐Au coordination is in place, but as the electrode separation is reduced, full coordination is achieved through Pt(II)‐Au interaction, and the conductance is greatly improved.

In order to fully rationalise the observed behaviour, we performed transport calculations by obtaining the ground state geometry for all molecules using the SIESTA implementation of Density Functional Theory (DFT).^[38]^ The optimised structures were then placed between Au electrodes, and the whole structure is relaxed to obtain the *device* ground state geometry. The Hamiltonian of each structure is thereby obtained, and we then used the transport code GOLLUM^[39]^ to calculate the transmission coefficient $T\left(E\right)$ for electrons of energy *E* transversing through each structure, using the Green's function method. We then used the Landauer formula to obtain the room‐temperature conductance, in units of $G/G_{0}$ .^[40]^

**Figure 4:**
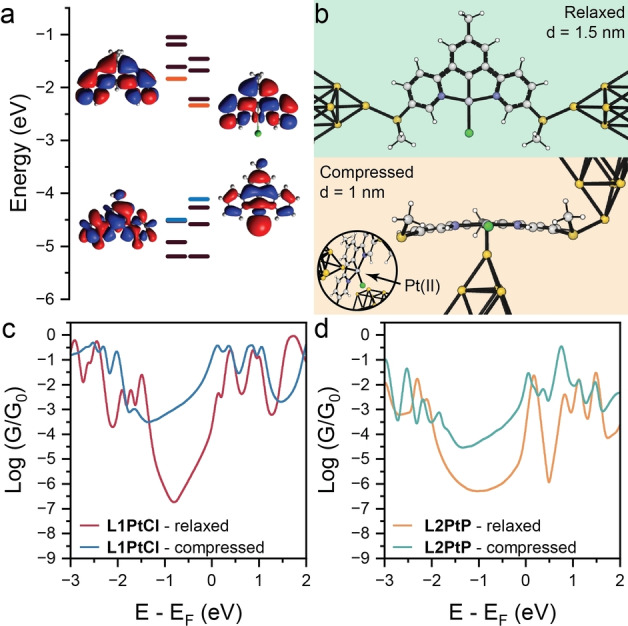
DFT calculations. a) Energy diagram for L1 and L1PtCl. HOMO and LUMO levels are highlighted, respectively, in blue and orange. b) Junction geometry with L1PtCl assembled between Au electrodes in the relaxed and compressed configurations. A close‐up view of the Au‐Pt(II) interface is shown in the inset. The distance between the apical Au atoms of the electrode is defined as *d*. These structures have been used to calculate the theoretical conductance (c) as a function of the Fermi energy for L1PtCl, in the two distinct configurations. d) Results of the same calculations performed on L2PtP.

We first focussed our efforts on understanding how complexation with Pt(II) affects the charge‐transport properties of the molecular wire. Figure 4a shows the frontier energy orbitals – Highest Occupied Molecular Orbital (HOMO) and Lowest Unoccupied Molecular Orbital (LUMO) – for the bare ligand L1 and its Pt(II) complex L1PtCl. It is immediately clear from analysis of the wavefunction spatial distribution that delocalisation of the wavefunction on the Pt(II) centre raises the HOMO energy and lowers the LUMO energy, shrinking the HOMO‐LUMO gap. This increases the mid‐gap transmission amplitude for a wide range around the DFT Fermi energy $E_{F}$ (see SI for further details). Similar phenomena are observed in the L2 series, with results shown in the SI along with additional details on wavefunction calculation and relative transmission curves. We then turned our attention to modelling the modulation of electrical conductance as a function of mechanical compression. As discussed earlier, our hypothesis is that as the electrodes are compressed, an increased interaction between the Pt(II) centre and a metallic electrode results in an increase in conductance. In order to model this, we considered the geometries shown in Figure 4b, with the apical Au atom of the electrode either coordinating to the methyl thioether terminus in the relaxed configuration, or directly to the Pt(II) centre in the compressed configuration. As can be observed in Figure 4c, the two geometries returned very different conductance values, and at the DFT Fermi level the predicted conductance enhancement is ~80, in good agreement with the experimental values. The same modelling performed on L2PtP (Figure 4d) was also able to correctly interpret our data, predicting an increase in conductance of approximately three orders of magnitude, and also predict the same phenomena operative in L2PtT. We were unable to obtain experimental data for the latter, due to the low‐conductance signal being too close to the noise level of the instrument (see SI for details). Our modelling and its excellent agreement with the experimental results strengthen our claim of the Au‐Pt(II) interaction being responsible for the observed conductance increase upon junction compression.

## Conclusions

We demonstrated here a novel method to introduce mechanoresistive phenomena in a single‐molecule junction, by exploiting the interaction between a Pt(II) ion and the Au electrode. We designed and synthesised two different series of molecular wires with a cyclometalated Pt(II) centre, where the contact point of the electrode can be mechanically switched in a reproducible and reliable way from a methyl thioether functional group, returning a low value of conductance, to the Pt(II) organometallic centre, associated with very efficient charge transport. With these compounds, we achieved amplitude of conductance modulation amongst the highest ever reported ($\frac{G_{HIGH}}{G_{LOW}}≅2700)$ , more than one order of magnitude larger than what is usually found with purely organic compounds,[^14^, ^17^, ^20^] and surpassing even those offered by non‐bonding interactions.[^19^, ^41^] The proposed mechanism for conductance switching was verified by a series of control experiments and DFT transport calculations on fully extended and compressed junctions. With these results, we expand the experimental toolbox of molecular electronics with another conceptually simple but very effective way to impart mechanoresistivity to a single‐molecule junction, exploiting previously unreported phenomena at the molecule‐electrode interface.

## Data Availability

All raw data acquired in Liverpool (NMR, Mass Spectrometry, STMBJ) can be accessed for free on the University of Liverpool Data Catalogue at DOI: 10.17638/datacat.liverpool.ac.uk/2482.

## Supplementary Information

Methods, experimental details, additional data and supporting calculations can be found in the supplementary information.

## Conflict of Interests

We declare no conflicts of interest.

## Supporting information

As a service to our authors and readers, this journal provides supporting information supplied by the authors. Such materials are peer reviewed and may be re‐organized for online delivery, but are not copy‐edited or typeset. Technical support issues arising from supporting information (other than missing files) should be addressed to the authors.

Supporting Information
