# Supplementary material for: Single‐Molecule Mechanoresistivity by Intermetallic Bonding
Source: Angew Chem Int Ed Engl. 2024 Nov 6;64(6):e202418062. doi: 10.1002/anie.202418062 (PMC11795739; doi:10.1002/anie.202418062)
Supplement: Supplementary file 1 — Supporting Information [file ANIE-64-e202418062-s001.pdf]

Angewandte Chemie
Eine Zeitschrift der Gesellschaft Deutscher Chemiker

Supporting Information

Single-Molecule Mechanoresistivity by Intermetallic Bonding

A. Sil, C. E. Spano, Y. Chelli, S. J. Higgins, S. Sangtarash, G. Piccinini, M. Graziano, R. J. Nichols, H. Sadeghi, A. Vezzoli**

Single-Molecule Mechanoresistivity by Intermetallic Bonding

Amit Sil,[a,#] Chiara E. Spano,[a,b,#] Yahia Chelli,[c,#] Simon J. Higgins,[a] Sara Sangtarash,[c] Richard J. Nichols,[a] Mariagrazia Graziano,[b] Hatef Sadeghi[c,*] & Andrea Vezzoli [a,*]

a) Department of Chemistry, University of Liverpool, Crown Street, Liverpool L69 7ZD, UK

b) Department of Electronics and Telecommunications, Politecnico di Torino, Corso Duca degli Abruzzi, 10129 Torino, Italy

c) Device Modelling Group, School of Engineering, University of Warwick, Coventry CV4 7AL, United Kingdom

#: these authors contributed equally to this work

* corresponding authors: andrea.vezzoli@liverpool.ac.uk

hatef.sadeghi@liverpool.ac.uk

Contents

1. Synthetic Details 3

1.1. Synthesis of **L1** and **L1PtCl** 3

1.2. Synthesis of **L2**, **L2PtCl**, **L2PtT** and **L2PtP** 5

1.3 Synthesis of **L3**, **L3PtCl** and **L3PtP** 9

2. NMR Spectra 12

3. Details on the STMBJ technique and additional STMBJ Data 23

3.1 Technical details 23

3.2 Data Analysis 24

3.3 Additional STMBJ Data 25

3.4 Break-Off Analysis 28

3.5 Data Tables 30

4. DFT Calculations 31

4.1. Methods 31

4.2 Additional DFT Calculations 31

4.2 DFT orbitals 33

References 35

1. Synthetic Details

Reagents were purchased from Merck, TCI UK or Fluorochem, depending on availability. Solvents were purchased from ThermoFisher scientific. Anhydrous solvents were obtained from a solvent purification system (Innovative Technology Puresolv) and stored over molecular sieves prior to use.

NMR spectra were recorded on a Bruker Avance III HD spectrometer. Chemical shifts δ are reported in ppm, referenced against the residual solvent and internal TMS standard. Flash chromatography was performed on Silica gel 40-63 μm (230-400 mesh, Merck). Mass spectrometry was carried out on an Agilent QTOF 7200 in EI or CI ionisation.

1.1. Synthesis of **L1** and **L1PtCl**

The synthetic routes to compound **L1PtCl** and **L1PtP** are shown in Scheme S1 below. First, compound **1** was synthesized in excellent yield (91%) starting from 1,3-dibromo-5-(tert-butyl)benzene which reacted with bis(pinacolato)diboron under Suzuki-Miyaura borylation conditions. **1** was then coupled 2-bromo-5-(methylthio)pyridine under Suzuki conditions to yield the desired ligand, 6,6'-(5-(tert-butyl)-1,3-phenylene)bis(3-(methylthio)pyridine) (**L1**) in good yield (87 %). **L1** was refluxed in degassed acetic acid with K_2PtCl_4 to afford the platinum complex **L1PtCl** (41 %).

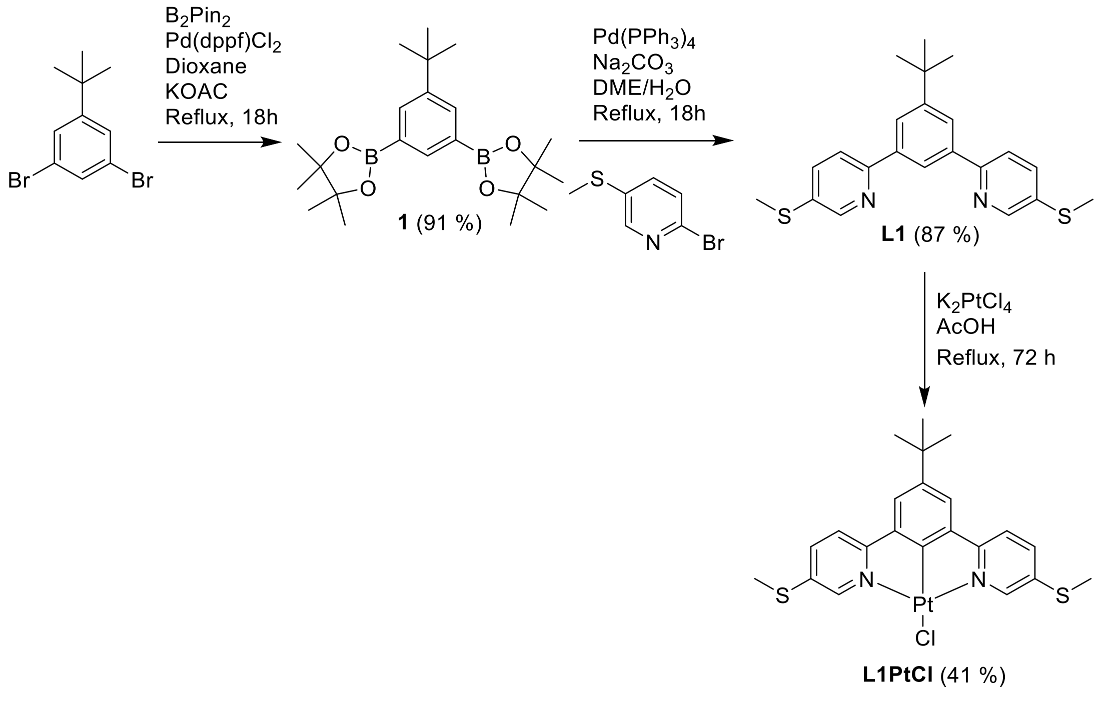

Scheme S1: Synthetic route for **L1** and **L1PtCl.**

Synthesis of 2,2'-(5-(tert-butyl)-1,3-phenylene)bis(4,4,5,5-tetramethyl-1,3,2-dioxaborolane) (1)

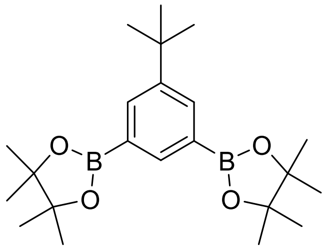

In a flame-dried 100 mL Schlenk flask, 1,3-dibromo-5-(tert-butyl)benzene (1.0 g, 3.43 mmol), bis(pinacolato)diboron (1.92 g, 7.53 mmol), KOAc (2.02 g, 20.6 mmol) and anhydrous 1,4-dioxane (35 mL) were added, and the mixture was degassed by three freeze-pump-thaw cycles. [1,1′-Bis(diphenylphosphino)ferrocene]dichloropalladium(II) (251 mg, 0.34 mmol) was added under a flow of nitrogen and the reaction mixture heated to 80 °C overnight under N_2 atmosphere. The mixture was then allowed to cool to room temperature, and the solvent was removed to isolate the crude intermediate which was purified by flash column chromatography on silica gel (hexanes:ethyl acetate, 30:70 *v:v*) to give compound **1** as a white solid (1.21 g, 91% yield). ^{1}H NMR (500 MHz, $CDCl_3$) δ 8.11 (s, 1H), 7.92 (d, *J* = 1.0 Hz, 2H), 1.34 (s, 24H).; ^{13}C NMR (126 MHz, $CDCl_3$) δ 149.5, 139.0, 134.7, 83.8, 34.9, 31.7, 25.1.; HRMS (CI) *m/z* = 387.2872 $[M+H]^+$; calculated for $[C_{22}H_{37}B_2O_4]^+$ 387.2878.

Synthesis of 6,6'-(5-(tert-butyl)-1,3-phenylene)bis(3-(methylthio)pyridine) (L1)

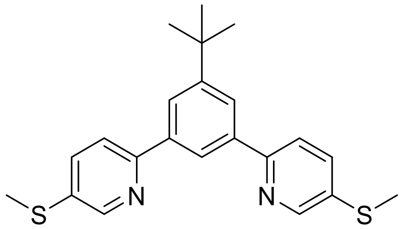

1 (0.5 g, 1.29 mmol), 2-bromo-5-(methylthio)pyridine (582 mg, 2.84 mmol), and Na_2CO_3 (1.09 g, 10.32 mmol) were suspended in a mixture of ethylene glycol dimethyl ether (DME)/water (11 mL/11 mL) and degassed by three freeze-pump-thaw cycles. $Pd(PPh_3)_4$ (75 mg, 0.06 mmol) was added under a flow of nitrogen and the reaction mixture was stirred at 85 °C overnight under N_2 atmosphere. The mixture was then allowed to cool to room temperature and all the volatiles were removed *in vacuo*. The residue was extracted with dichloromethane, washed with brine, and dried over anhydrous magnesium sulfate. After filtration and evaporation of the solvent, the crude product was purified by column chromatography over silica gel (hexanes:ethyl acetate, 75:25 *v:v*) as eluent to afford **L1** as an off-white solid (428 mg, 87 % yield). ^{1}H NMR (500 MHz, CD_2Cl_2) δ 8.70 – 8.48 (m, 2H), 8.38 (t, *J* = 1.5 Hz, 1H), 8.07 (d, *J* = 1.5 Hz, 2H), 7.77 (dd, *J* = 8.4, 0.6 Hz, 2H), 7.66 (dd, *J* = 8.4, 2.4 Hz, 2H), 2.54 (s, 6H), 1.43 (s, 9H). ^{13}C NMR (126 MHz, CD_2Cl_2) δ 154.2, 152.3, 147.7, 139.1, 134.9, 134.1, 124.2, 122.2, 120.4, 35.1, 31.3, 15.8.; HRMS (CI) *m/z* = 381.1458 $[M+H]^+$; calculated for $[C_{36}H_{35}BrN_2O]^+$ 381.1459

Synthesis of L1PtCl

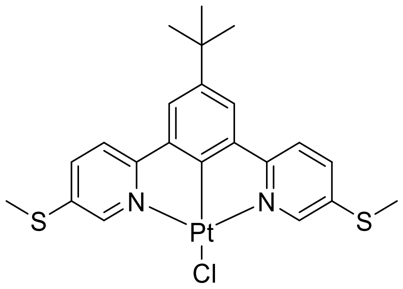

A mixture of **L1** (166 mg, 0.44 mmol), K_2PtCl_4 (206 mg, 0.49 mmol) and acetic acid (20 mL) were degassed by three freeze-pump-thaw cycles. The reaction mixture was then heated at reflux (118 °C) for 72 h under nitrogen before cooling to room temperature. Water (5 mL) was added, and the solid product was separated in a centrifuge; the crude product was then washed with water, methanol, and diethyl ether. The residue was extracted into dichloromethane and the solvent removed under reduced pressure to give the desired complex as yellow solid (107 mg, 41% yield). ^{1}H NMR (500 MHz, CD_2Cl_2) δ 9.13 (d, *J* = 1.9 Hz, 2H), 7.81 (dd,

J = 8.5, 2.2 Hz, 2H), 7.62 (d, *J* = 8.3 Hz, 2H), 7.48 (s, 2H), 2.59 (s, 6H), 1.40 (s, 9H).; ^{13}C NMR (126 MHz, CD_2Cl_2) δ 163.9, 149.3, 146.7, 140.3, 136.7, 136.0, 120.8, 119.0, 35.3, 31.4, 15.7. HRMS (CI) *m/z* = 574.0934 $[M\text{-}Cl]^+$; calculated for $[C_{22}H_{23}N_2PtS_2]^+$ 574.0950.

1.2. Synthesis of **L2**, **L2PtCl**, **L2PtT** and **L2PtP**

The synthetic route to our target platinum complexes **L2PtT** and **L2PtP** is shown below Scheme S2. Ligand **L2** was synthesised starting form 1,3,5 tribromobenzene by treatment with (4-(methylthio)phenyl)boronic acid under Suzuki conditions to yield **2** (59 %). Treatment of bis(pinacolato)diboron with compound **2** under Suzuki-Miyaura borylation conditions yielded the borylated derivative **3**, which was then reacted with 2-chloro-4-(tert-butyl)pyridine under Suzuki cross-coupling coditions to afford the desired ligand **L2** in excellent yield (90 %). Ligand **L2** was then reacted with K_2PtCl_4 in refluxed degassed acetic acid to achieve the key platinum intermediate **L2PtCl** (75 %). **L2PtCl** was treated with (4-ethynylphenyl)(methyl)sulfane[1] or 4-ethynylpyridine[2] in the presence of NaOMe to afford the desired platinum complexes **L2PtT** (48 %) and **L2PtP** (55 %), respectively.

(4-Ethynylphenyl)(methyl)sulfane and 4-ethynylpyridine were synthesized by Sonogashira coupling of the corresponding bromoderivative with trimethylsilylacetylene, followed by deprotection using potassium carbonate in a solvent mixture of methanol and dichloromethane, as discussed in the respective references.

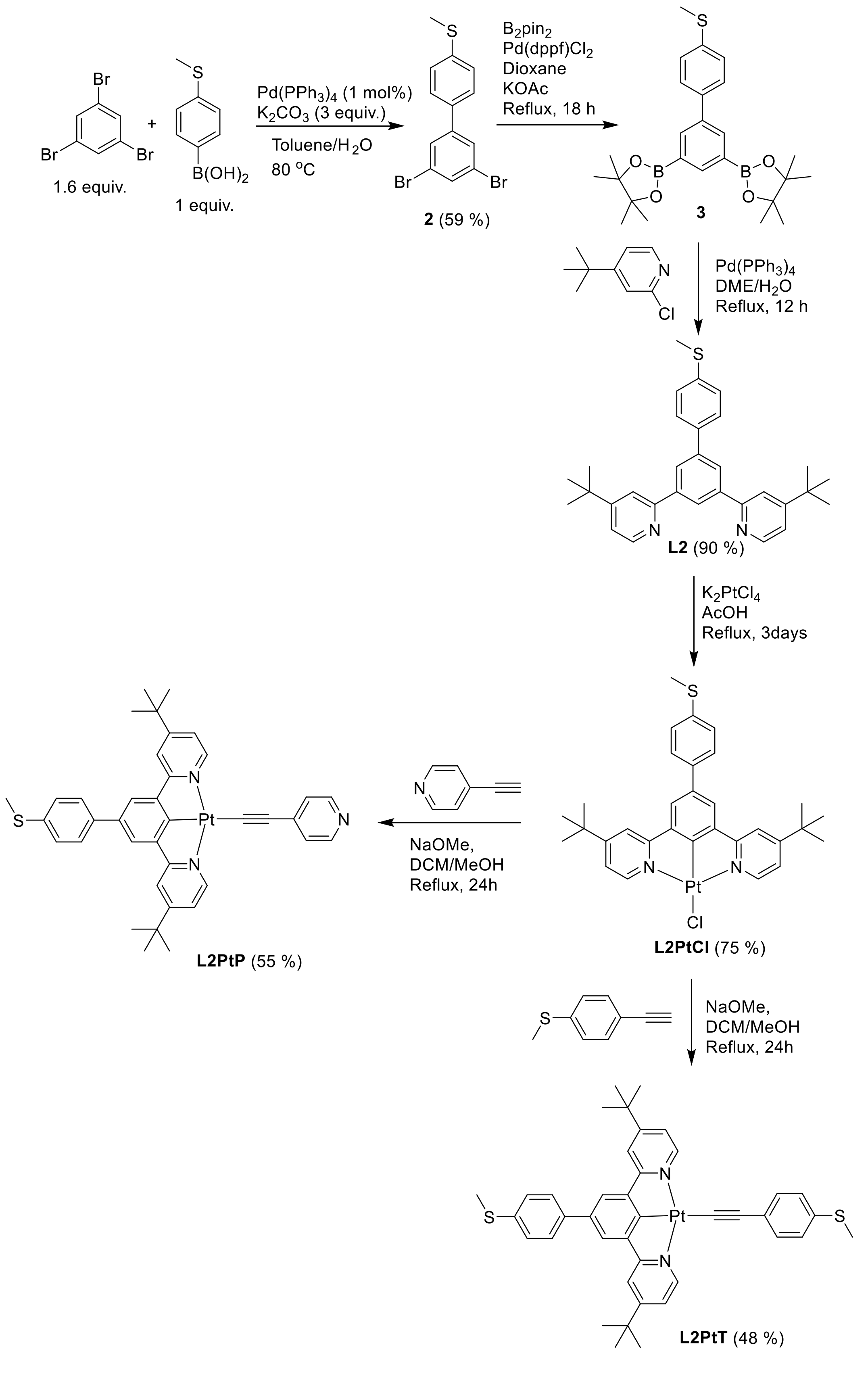

Scheme S2: Synthetic route to L**2**, **L2PtCl, L2PtT** and **L2PtP**

Synthesis of (3',5'-dibromo-[1,1'-biphenyl]-4-yl)(methyl)sulfane (2)

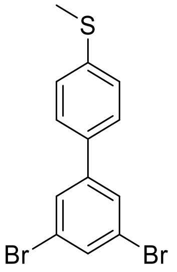

(4-(Methylthio)phenyl)boronic acid (1.0 g, 5.95 mmol), 1,3,5-tribromobenzene (2.32 g, 7.43 mmol) and K_2CO_3 (2.46 g, 17.85 mmol) were suspended in a mixture of toluene/water (23 mL/ 9 mL) in a 100 mL Schlenk flask and degassed using three freeze-pump-thaw cycles. $Pd(PPh_3)_4$ (103 mg, 0.09 mmol) was added under a flow of nitrogen and the reaction mixture heated to 85 °C overnight under N_2 atmosphere. After cooling, the crude mixture was washed with water and extracted into dichloromethane (3 × 30 mL) before drying over anhydrous $MgSO_4$. The solution was filtered and the solvent removed under reduced pressure before purification by column chromatography over silica gel (petroleum ether:dichloromethane, 95:5 *v:v*) as eluent to give the title compound **2** as a white solid (1.59 g, 59 %). ^{1}H NMR (500 MHz, $CDCl_3$) δ 7.64 – 7.59 (m, 3H), 7.48 – 7.41 (m, 2H), 7.35 – 7.29 (m, 2H), 2.52 (s, 3H). ^{13}C NMR (126 MHz, $CDCl_3$) δ 144.3, 139.7, 135.0, 132.6, 128.8, 127.6, 126.9, 123.5, 15.8.; HRMS (CI) *m/z* = 358.8922 $[M+H]^+$; calculated for $[C_{13}H_{11}Br_2S]^+$ 358.8928.

Synthesis of 2,2'-(4'-(methylthio)-[1,1'-biphenyl]-3,5-diyl)bis(4,4,5,5-tetramethyl-1,3,2-dioxaborolane) (3)

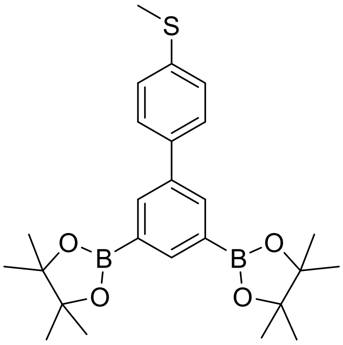

A mixture of **2** (600 mg, 1.67 mmol), bis(pinacolato)diboron (932 mg, 3.67 mmol), KOAc (983 mg, 10.02 mmol) and anhydrous 1,4-dioxane (20 mL) were degassed by three freeze-pump-thaw cycles. [1,1′-bis(diphenylphosphino)ferrocene]dichloropalladium(II) (122 mg, 0.17 mmol) was added under a flow of nitrogen and the reaction mixture heated to 80°C overnight under an atmosphere of nitrogen. The mixture was allowed to cool to room temperature and the solvent was removed to isolate the crude intermediate of compound **3** which was used without further purification.

Synthesis of 2,2'-(4'-(methylthio)-[1,1'-biphenyl]-3,5-diyl)bis(4-(tert-butyl)pyridine) (L2)

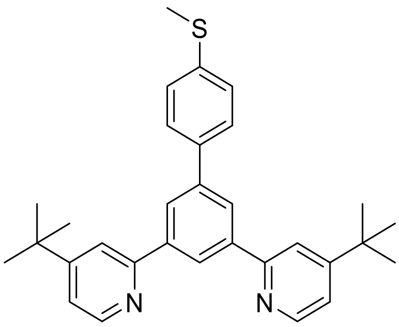

In a 100 ml Schlenk flask, compound **3** (755 mg, 1.67 mmol), 2-chloro-4-(tert-butyl)pyridine (566 mg, 3.34 mmol) and Na_2CO_3 was suspended in a mixture of DME/H_2O (14 mL/ 14mL) and degassed by three freeze-pump-thaw cycles. $Pd(PPh_3)_4$ (97 mg, 0.08 mmol) was added to this under a flow of N_2 atmosphere. The reaction mixture was heated to 80 °C overnight under an atmosphere of nitrogen. After cooling, the organic phase was washed with brine, water and dried over anhydrous $MgSO_4$. All the volatiles were removed under reduced pressure. The crude product was purified by column chromatography over silica gel with petroleum ether: ethyl acetate mixture (v:v, 70:30) as eluent to afford the title ligand **L2** as an off white solid (705 mg, 90 %). ^{1}H NMR (500 MHz, $CDCl_3$) δ 8.67 (d, *J* = 5.2 Hz, 2H), 8.52 (t, *J* = 1.6 Hz, 2H), 8.26 (d, *J* = 1.6 Hz, 2H), 7.86 (d, *J* = 1.3 Hz, 2H), 7.79 – 7.71 (m, 2H), 7.43 – 7.37 (m, 2H), 7.30 (dd, *J* = 5.3, 1.8 Hz, 2H), 2.54 (s, 3H), 1.41 (s, 18H).; ^{13}C NMR (126 MHz, $CDCl_3$) δ 161.0, 157.5, 149.7, 141.7, 141.3, 138.0,

138.0, 127.9, 127.0, 126.2, 124.9, 119.7, 118.2, 35.05, 30.8.; HRMS (CI) *m/z* = 467.2516 $[M+H]^+$; calculated for $[C_{31}H_{35}N_2S]^+$ 465.2521.

Synthesis of L2PtCl

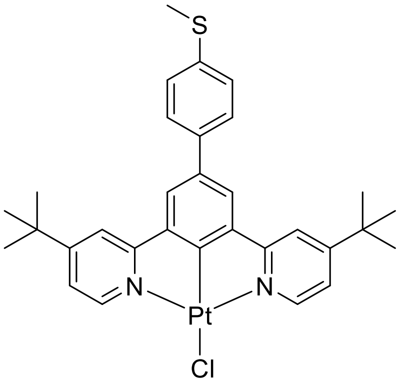

Potassium tetrachloroplatinate(II) (212 mg, 0.454 mmol) was added to a solution of **L1** in glacial acetic acid (22 mL) in a Schlenk flask and the solution was degassed using three freeze-pump-thaw cycles. The reaction mixture was then heated at reflux (118 °C) for 72 h under N_2 atmosphere before cooling to room temperature. Water (5 mL) was added, and the solid product was separated on a centrifuge. The solid fraction was separated and washed with water, methanol, and diethyl ether. The solid product was then extracted into dichloromethane and the solvent removed under reduced pressure to yellow solid product **L2PtCl** (238 mg, 75%). ^{1}H NMR (500 MHz, DMSO-d6) δ 8.98 (d, *J* = 6.1 Hz, 2H), 8.29 (d, *J* = 1.9 Hz, 2H), 8.23 (s, 2H), 7.89 (d, *J* = 8.4 Hz, 2H), 7.57 (dd, *J* = 6.1, 2.1 Hz, 2H), 7.41 (d, *J* = 8.4 Hz, 2H), 2.55 (s, 3H), 1.42 (s, 18H).; ^{13}C NMR (126 MHz, DMSO-d6) δ 166.6, 164.9, 160.2, 151.1, 141.7, 137.9, 137.3, 135.5, 127.7, 126.9, 123.6, 121.4, 118.5, 36.1, 30.5. HRMS (CI) *m/z* = 660.2003 $[M-Cl]^+$; calculated for $[C_{31}H_{33}N_2PtS]^+$ 660.2012.

Synthesis of L2PtT

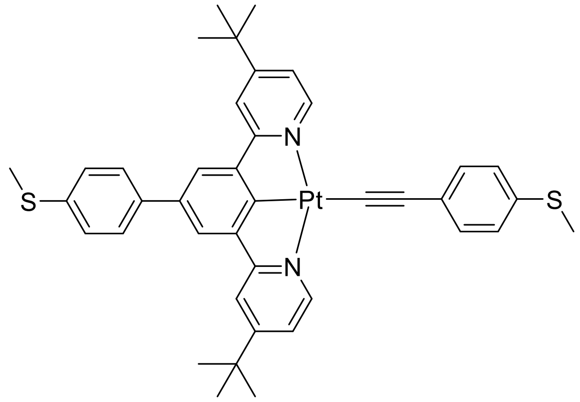

In an oven dried 100 mL Schlenk flask purged with N_2, (4-ethynylphenyl)(methyl)sulfane (31 mg, 0.287 mmol) and sodium methoxide (16 mg, 0.287 mmol) were stirred in dry methanol (20 mL) at room temperature for half an hour and **L2PtCl** (100 mg, 0.143 mmol) in dichloromethane (20 mL) was then added. The temperature was raised to 60 °C and stirring was continued for 48 h. Upon cooling to room temperature, the solvent was removed in *vacuo* and the resulting orange solid was washed with methanol and diethyl ether, and extracted in dichloromethane. Recrystallisation from dichloromethane/hexane under inert atmosphere afforded **L2PtT** as orange solid (56 mg, 48 %);^{1}H NMR (500 MHz, $CDCl_3$) δ 9.34 (d, *J* = 6.1 Hz, 2H), 7.69 (d, *J* = 2.0 Hz, 2H), 7.66 (s, 2H), 7.61 (d, *J* = 8.3 Hz, 2H), 7.50 (d, *J* = 8.3 Hz, 2H), 7.39 (d, *J* = 8.3 Hz, 2H), 7.23 – 7.17 (m, 2H), 2.55 (s, 3H), 2.50 (s, 3H), 1.41 (s, 18H).; ^{13}C NMR (126 MHz, $CDCl_3$) δ 177.7, 169.1, 163.5, 155.1, 144.1, 139.7, 137.3, 136.5, 134.7, 132.3, 127.8, 127.5, 126.9, 125.8, 122.7, 121.0, 116.6, 111.8, 35.7, 30.6. HRMS (ASAP) *m/z* = 807.2319 $[M+H]^+$; calculated for $[C_{40}H_{41}N_2S_2{}^{194}Pt]^+$ 807.2338.

Synthesis of L2PtP

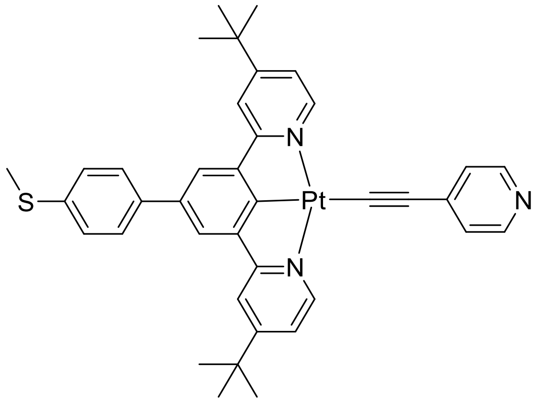

Compound **L1PtP** was synthesized following a similar procedure to that employed to prepare **L2PtT**. 4-ethynylpyridine (15 mg, 0.15 mmol) and sodium methoxide (11 mg, 0.02 mmol) were stirred in dry methanol (8 mL) at room temperature for half an hour and **L2PtCl** (70 mg, 0.10 mmol) in dichloromethane (8 mL) was then added. The temperature was raised to 60 °C and stirring was continued for 48 h. Upon cooling to room temperature, the solvent was removed in *vacuo* and the resulting yellow solid was washed with methanol and diethyl ether, and extracted in dichloromethane. Recrystallisation from dichloromethane/hexane under inert atmosphere afforded pure **L2PtP** (42 mg, 55 %); ^{1}H NMR (500 MHz, CD_2Cl_2) δ 9.24 (d, *J* = 6.1 Hz, 2H), 8.48 (dd, *J* = 4.5, 1.6 Hz, 2H), 7.84 – 7.78 (m, 2H), 7.74 – 7.66 (m, 2H), 7.46 – 7.41 (m, 2H), 7.38 (dd, *J* = 4.5, 1.6 Hz, 2H), 7.30 (dd, *J* = 6.1, 2.1 Hz, 2H), 2.59 (s, 3H), 1.46 (s, 18H).; ^{13}C NMR (126 MHz, CD_2Cl_2) δ 176.6, 168.8, 163.9, 154.5, 149.3, 145.9, 144.0, 138.9, 137.5, 136.4, 136.3, 127.4, 126.9, 125.9, 122.5, 121.2, 116.9, 109.0, 67.7, 35.5, 30.1.; HRMS (CI) *m/z* = 763.2438 $[M+H]^+$; calculated for $[C_{38}H_{38}N_3PtS]^+$ 763.2434.

1.3 Synthesis of **L3**, **L3PtCl** and **L3PtP**

The synthetic route to the target Pt(II) species **L3PtP** is shown below in Scheme 4. Ligand **L3** was synthesised starting form 1,3-phenylenediboronic acid and 2-chloro-4-(tert-butyl)pyridine following by Suzuki cross coupling method. Ligand **L3** was then reacted with K_2PtCl_4 in refluxed degassed acetic acid to give the key Pt(II) precursor **L3PtCl** in good yield (72 %). **L3PtCl** was further reacted with 4-ethynylpyridine in presence of NaOMe to afford the desired compound **L3PtP** (41 %) in moderate yield.

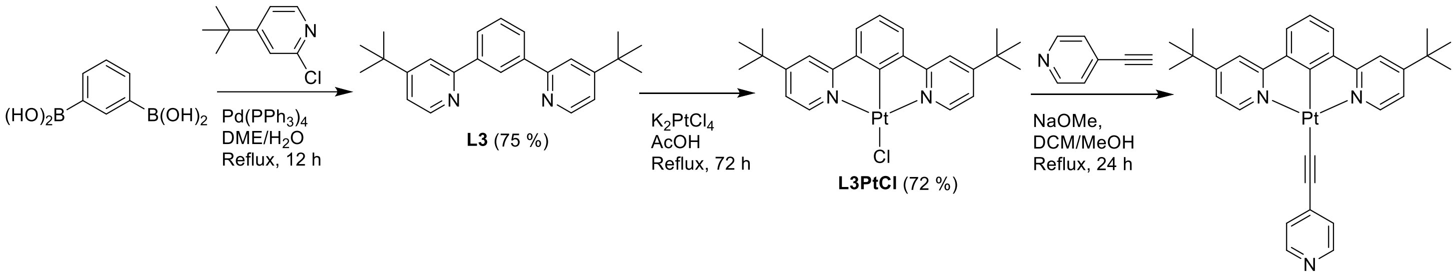

Scheme S3:: Synthetic route for L**3**, **L3PtCl,** and **L3PtP**

Synthesis of 1,3-bis(4-(tert-butyl)pyridin-2-yl)benzene (L3)

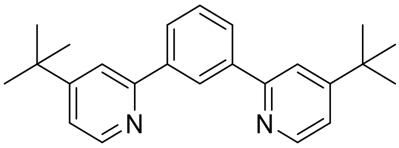

In a 100 ml Schlenk flask, 1,3-phenylenediboronic acid (200 mg, 1.20 mmol), 2-chloro-4-(tert-butyl)pyridine (490 mg, 2.89 mmol) and Na_2CO_3 were suspended in a mixture of DME/H_2O (9.5 mL/ 9.5 mL) and degassed by three freeze-pump-thaw cycles. $Pd(PPh_3)_4$ (70 mg, 0.06 mmol) was then added under N_2 atmosphere. The reaction mixture was heated to 80 °C overnight. After cooling, the organic phase was washed with brine, water, and dried over anhydrous $MgSO_4$. All the volatilities were removed under reduced pressure. The crude product was purified by column chromatography over silica gel with petroleum ether:ethyl acetate (v:v, 85:15) as eluent to afford the title ligand **L3** as a light yellow oil (313 mg, 75 %). ^{1}H NMR (500 MHz, Chloroform-*d*) δ = 8.62 (d, *J* = 5.3 Hz, 2H), 8.54 (s, 1H), 8.01 (dd, *J* = 7.8, 1.8 Hz, 2H), 7.79 (d, *J* = 1.7 Hz, 2H), 7.59 (t, *J* = 7.7 Hz, 1H), 7.26 (dd, *J* = 5.2, 2.0 Hz, 2H), 1.38 (s, 18H); ^{13}C NMR (126 MHz, CDCl3) δ = 161.0, 157.7, 149.7, 140.8, 129.3, 127.7, 126.1, 119.7, 118.3, 35.1, 30.8; HRMS (ESI+) *m/z* = 345.2330 $[M+H]^+$; calculated for $[C_{24}H_{29}N_2]^+$ 345.2331.

Synthesis of L3PtCl

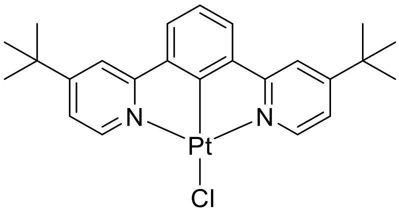

Potassium tetrachloroplatinate(II) (138 mg, 0.33 mmol) was added to a solution of compound **L3** (100 mg, 0.29 mmol) in glacial acetic acid (13 mL) in a Schlenk flask and the solution was degassed using three freeze-pump-thaw cycles. The reaction mixture was then heated at reflux (118 °C) for 72 h under nitrogen before cooling to room temperature. Water (5 mL) was added, and the solid product was separated by centrifugation. The crude product was washed with water, methanol, and diethyl ether. The residue was then extracted into DCM and the solvent removed under reduced pressure to give **L3PtCl** as a yellow-green solid (120 mg, 72%). ^{1}H NMR (500 MHz, DMSO-d_6) δ = 8.97 (d, *J* = 6.1 Hz, 2H), 8.10 (d, *J* = 2.2 Hz, 2H), 7.90 (d, *J* = 7.7 Hz, 2H), 7.58 (dd, *J* = 6.2, 2.2 Hz, 2H), 7.29 (t, *J* = 7.7 Hz, 1H), 1.39 (s, 18H). HRMS (ESI+) *m/z* = 538.1822 $[M-Cl]^+$; calculated for $[C_{24}H_{27}N_2Pt]^+$ 538.1822. We were not able to record a ^{13}C NMR spectrum due to very low solubility in common deuterated solvents.

Synthesis of L3PtP

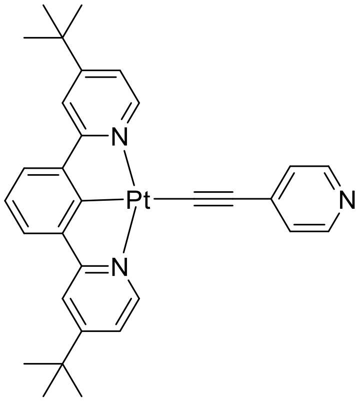

Compound **L3PtP** was synthesized following a similar procedure to that used for **L2PtT**. 4-ethynylpyridine (9 mg, 0.09 mmol) and NaOMe (7 mg, 0.129 mmol) were stirred in dry MeOH (5 mL) at RT for half an hour, and **L3PtCl** (25 mg, 0.043 mmol) in DCM (5 mL) was then added. The mixture was stirred at 60 °C for 48 h. After cooling to room temperature, the solvent was removed in *vacuo* and the resulting orange solid was washed with MeOH and Et_2O and extracted in dichloromethane. Recrystallisation from DCM/Hexane under inert atmosphere afforded **L3PtP** as a yellow solid (11 mg, 41 %); ^{1}H NMR (500 MHz, Chloroform-*d*) δ = 9.23 (d, *J* = 6.1 Hz, 2H), 8.55 – 8.36 (m, 2H), 7.65 (d, *J* = 2.1 Hz, 2H), 7.54 (d, *J* = 7.6 Hz, 2H), 7.42 – 7.33 (m, 2H), 7.21 (dt, *J* = 5.9, 3.4 Hz, 3H), 1.40 (s, 18H). HRMS (CI) *m/z* = 641.2248 $[M+H]^+$; calculated for $[C_{31}H_{32}N_3Pt]^+$ 641.2244. We were not able to record a ^{13}C NMR spectrum due to very low solubility in common deuterated solvents.

2. NMR Spectra

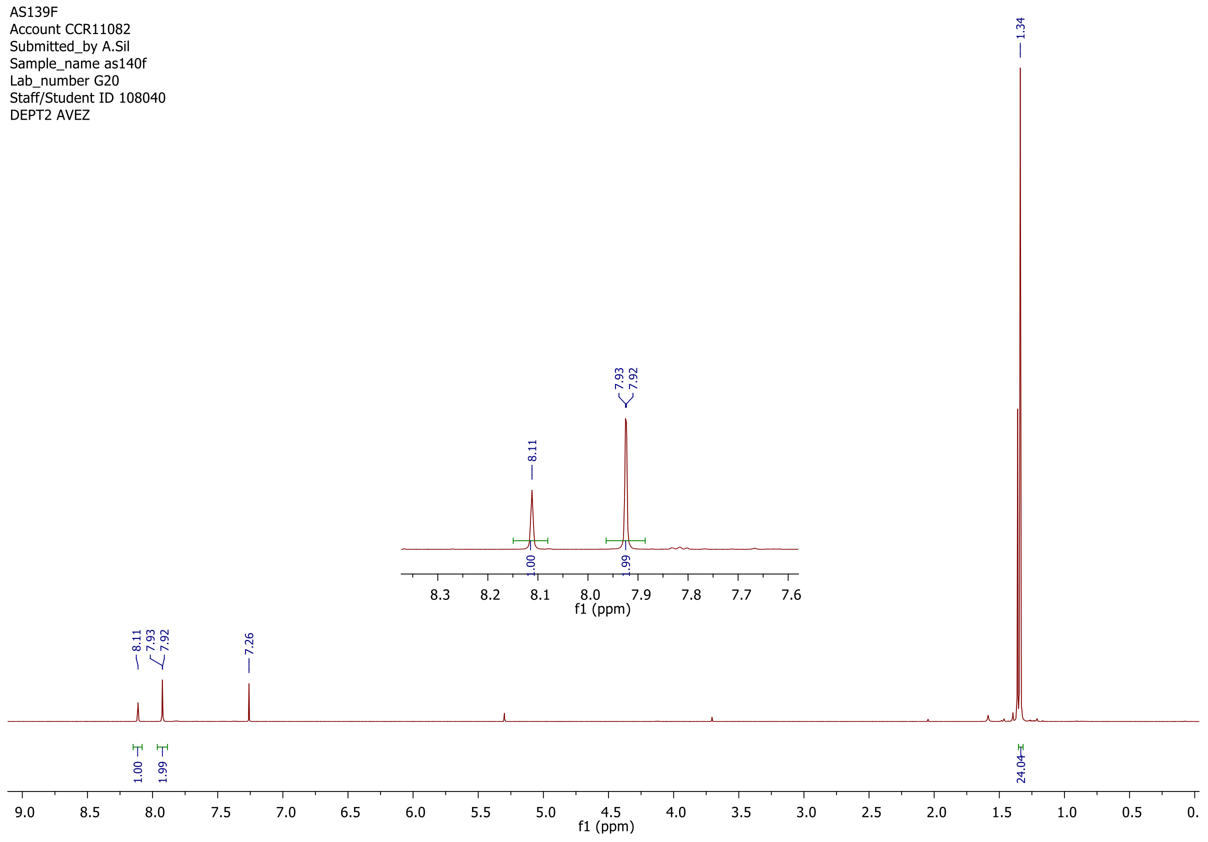

Figure S1: ^{1}H NMR (500 MHz, $CDCl_3$) of **1**

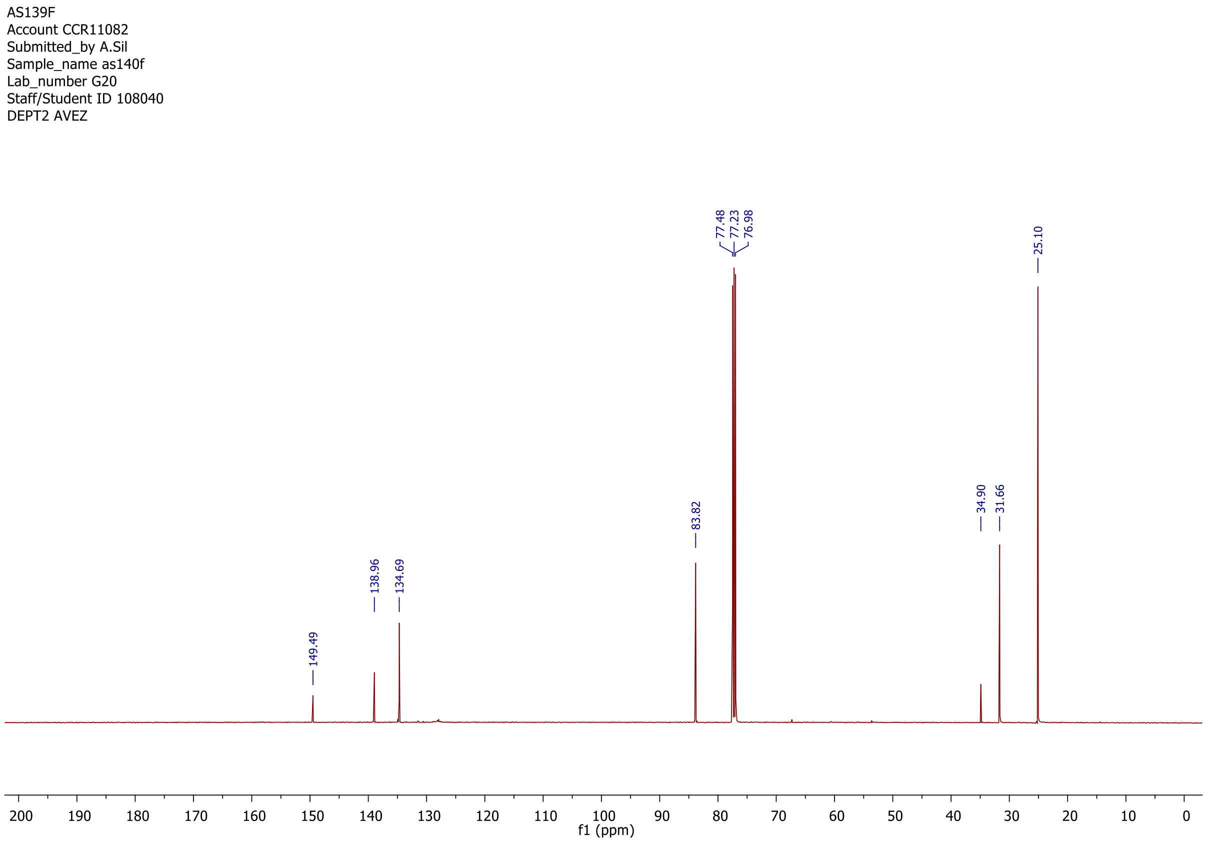

Figure S2: ^{13}C NMR (126 MHz, $CDCl_3$) of **1**

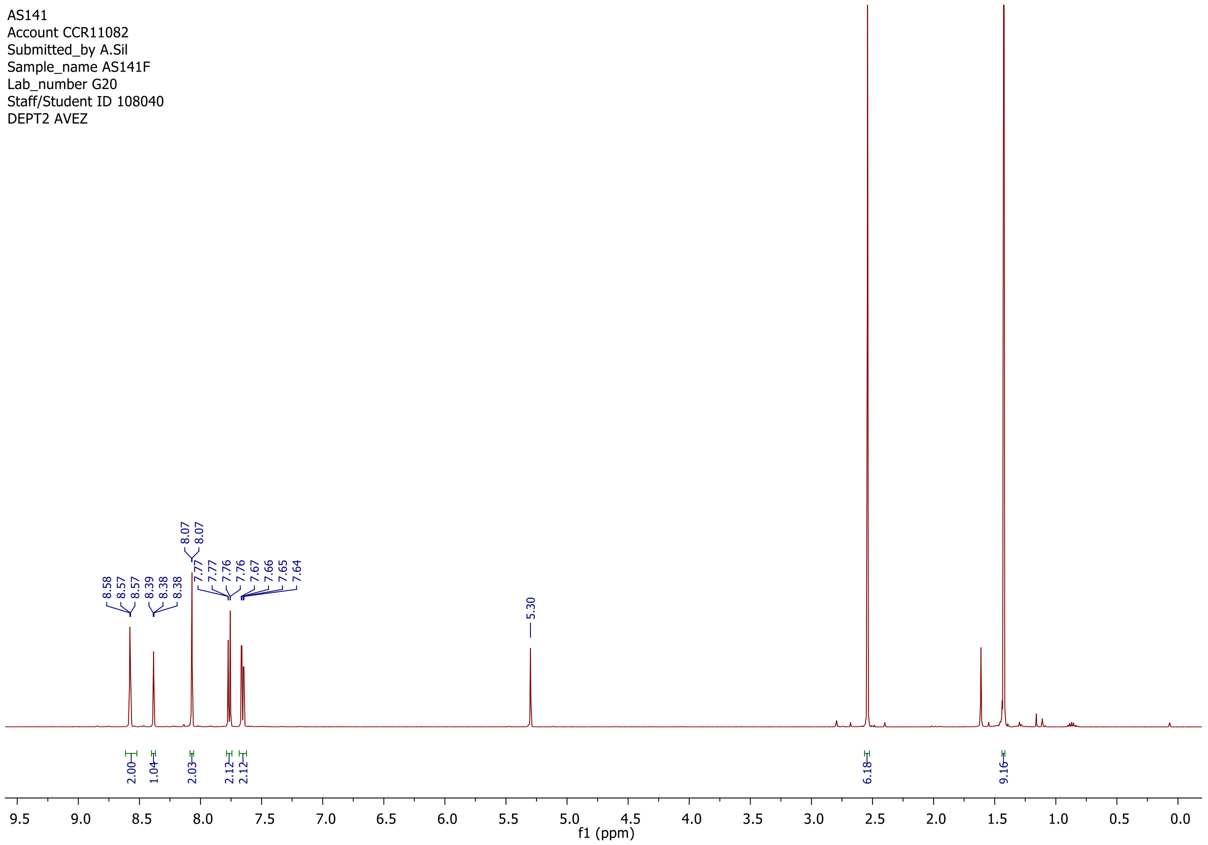

Figure S3: ^{1}H NMR (500 MHz, CD_2Cl_2) of **L1**

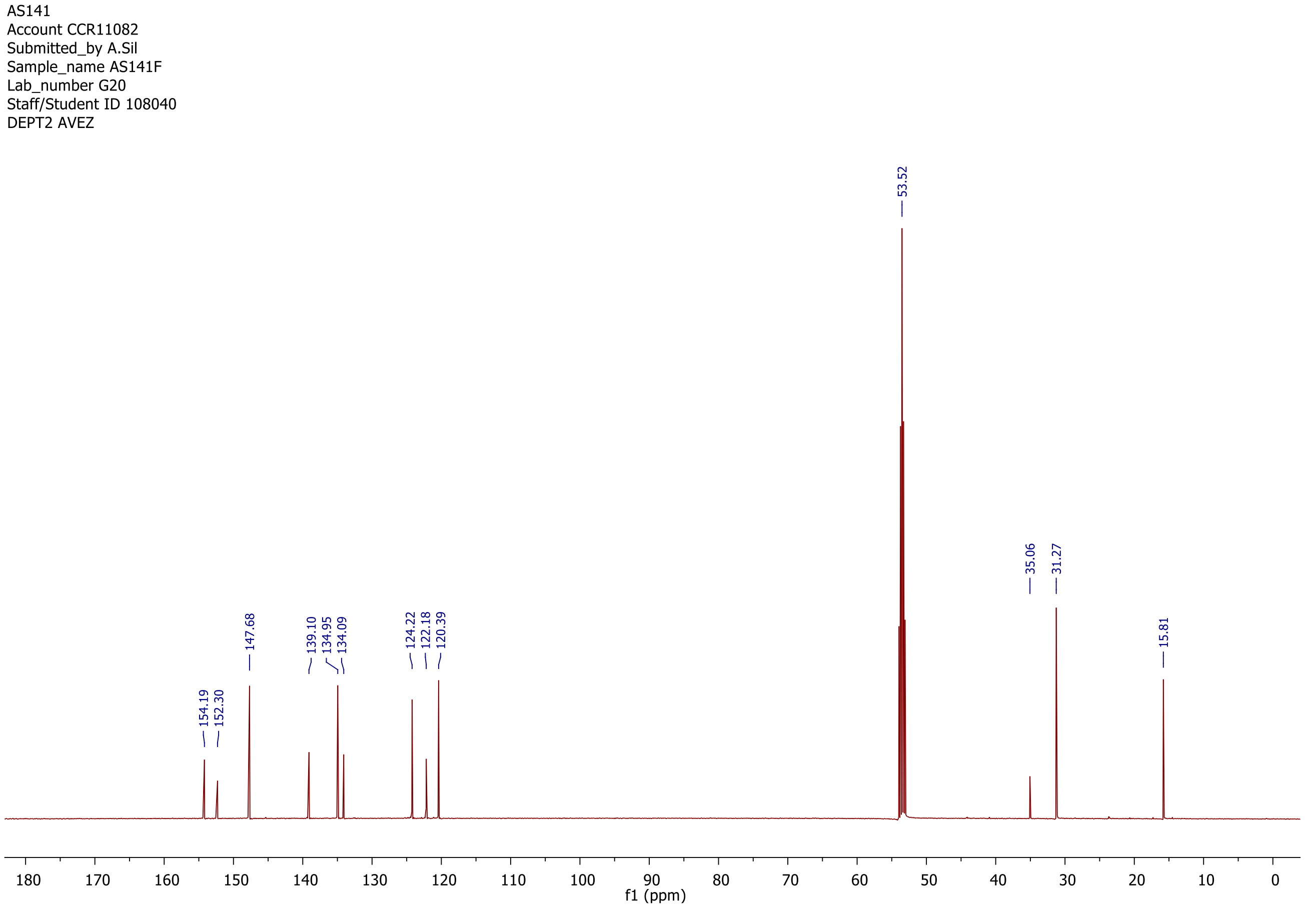

Figure S4: ^{13}C NMR (126 MHz, $CDCl_3$) of **L1**

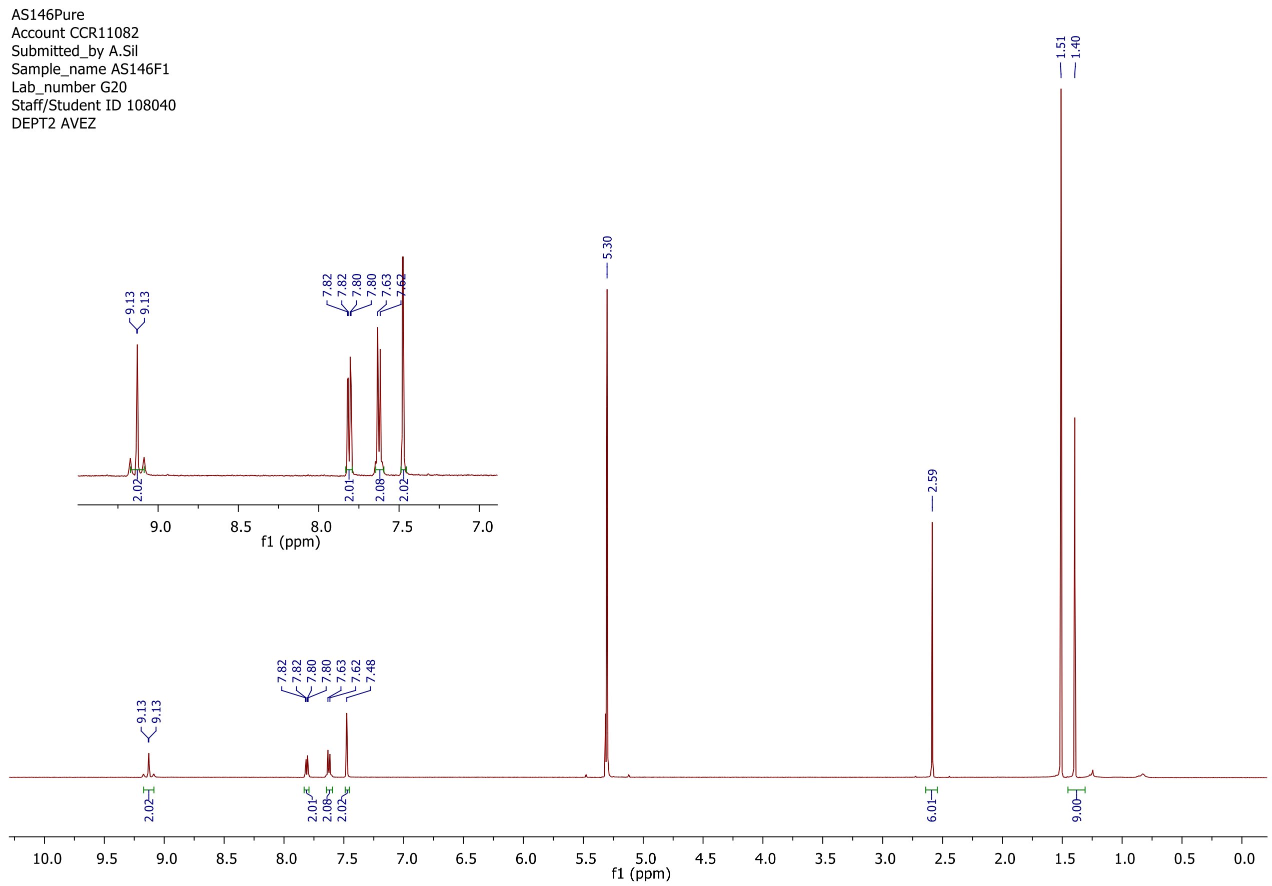

Figure S5: ^{1}H NMR (500 MHz, CD_2Cl_2) of **L1PtCl**

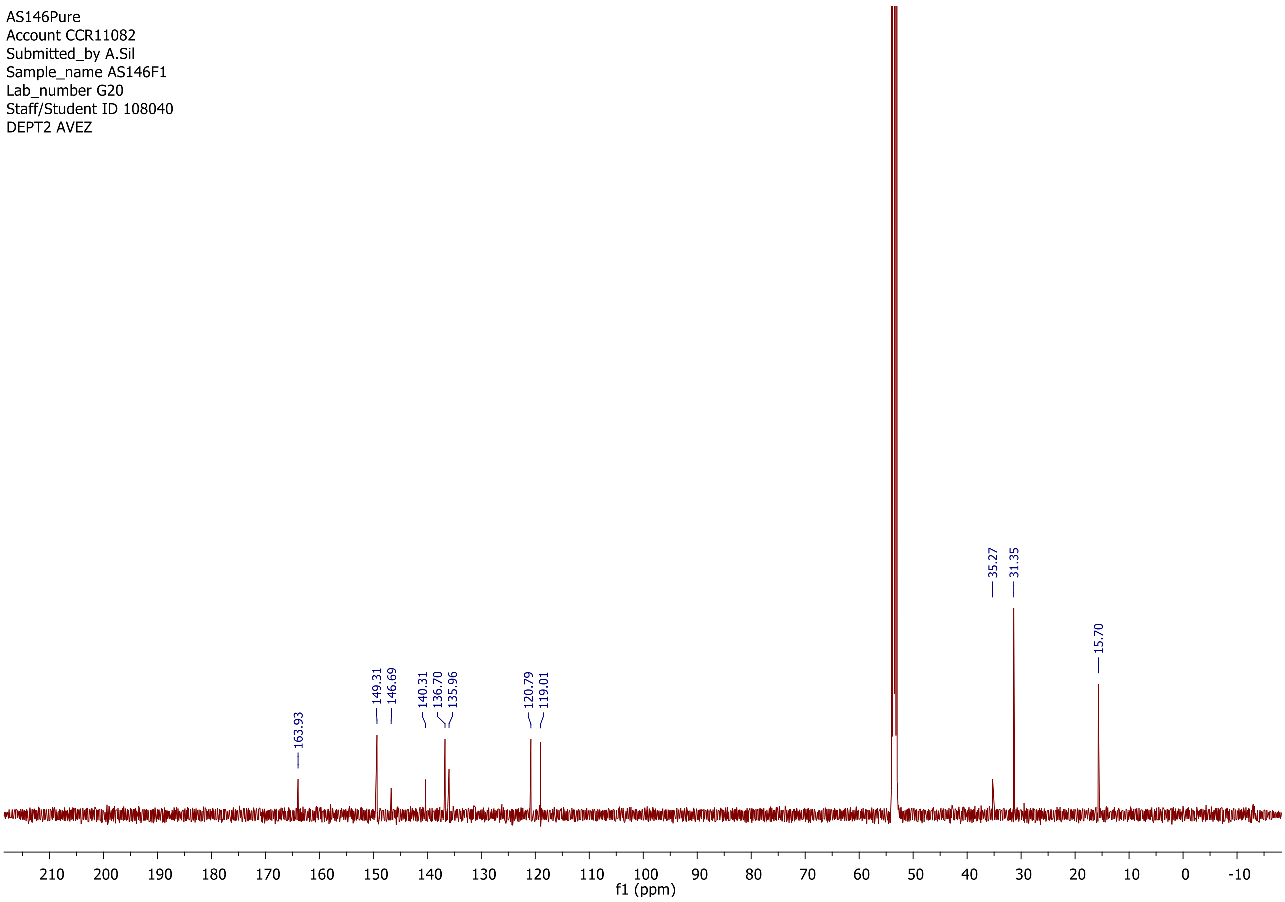

Figure S6: ^{13}C NMR (126 MHz, CD_2Cl_2) of **L1PtCl**

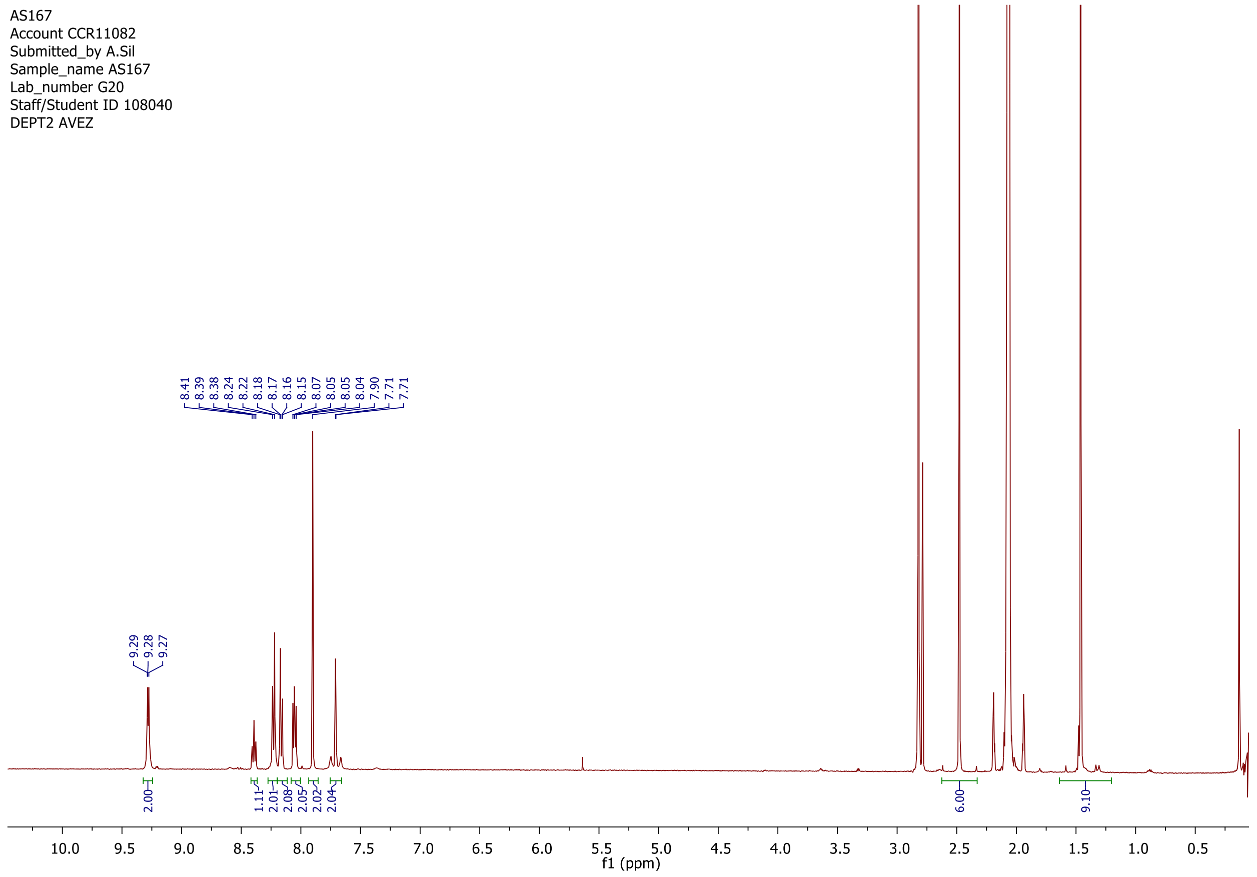

Figure S7: ^{1}H NMR (500 MHz, Acetone-*d6*) of **L1PtP**

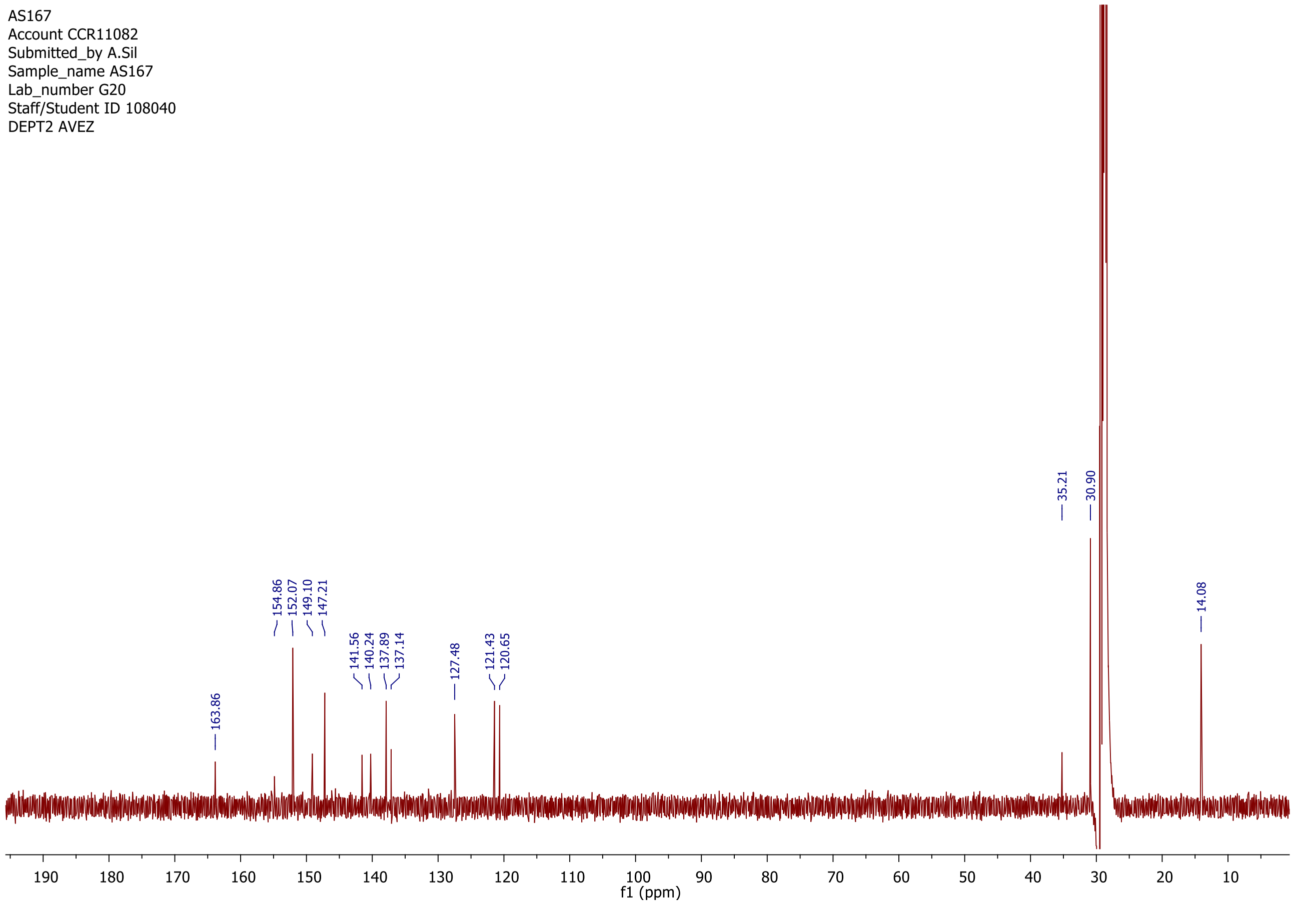

Figure S8: ^{13}C NMR (126 MHz, Acetone-*d6*) of **L1PtP**

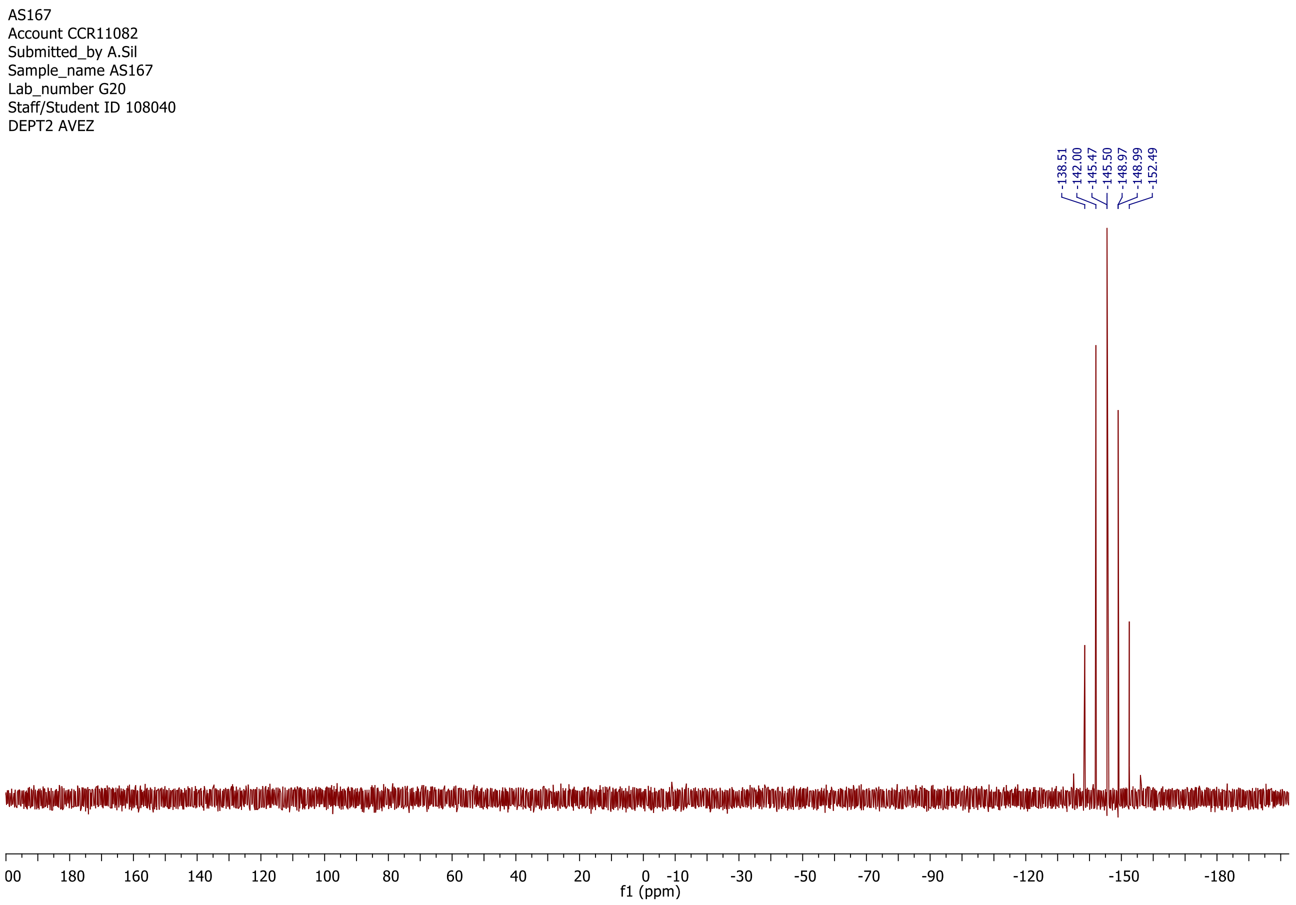

Figure S9: ^{31}P NMR (202 MHz, Acetone-d6) of **L1PtP**

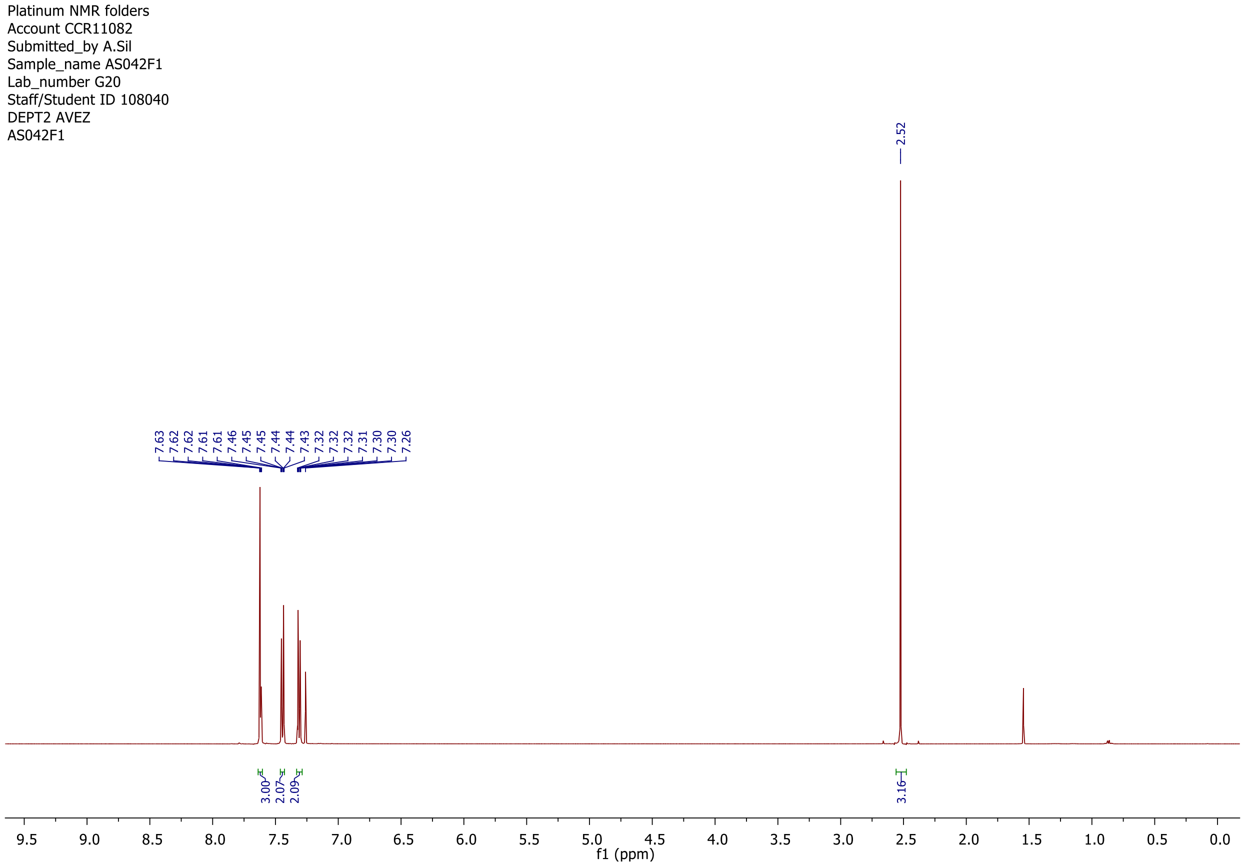

Figure S10: ^{1}H NMR (500 MHz, $CDCl_3$) of **2**

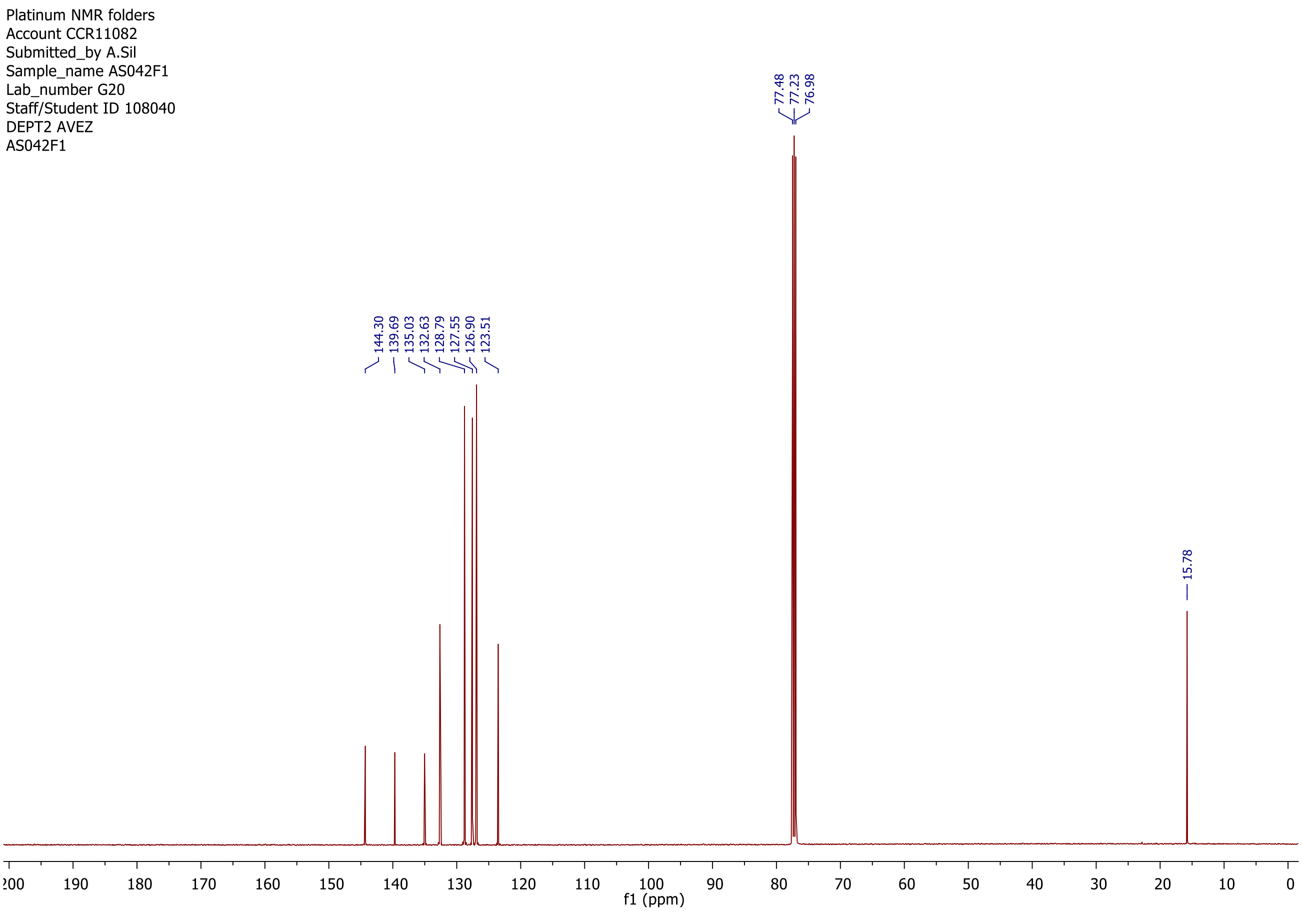

Figure S11: ^{13}C NMR (126 MHz, $CDCl_3$) of **2**

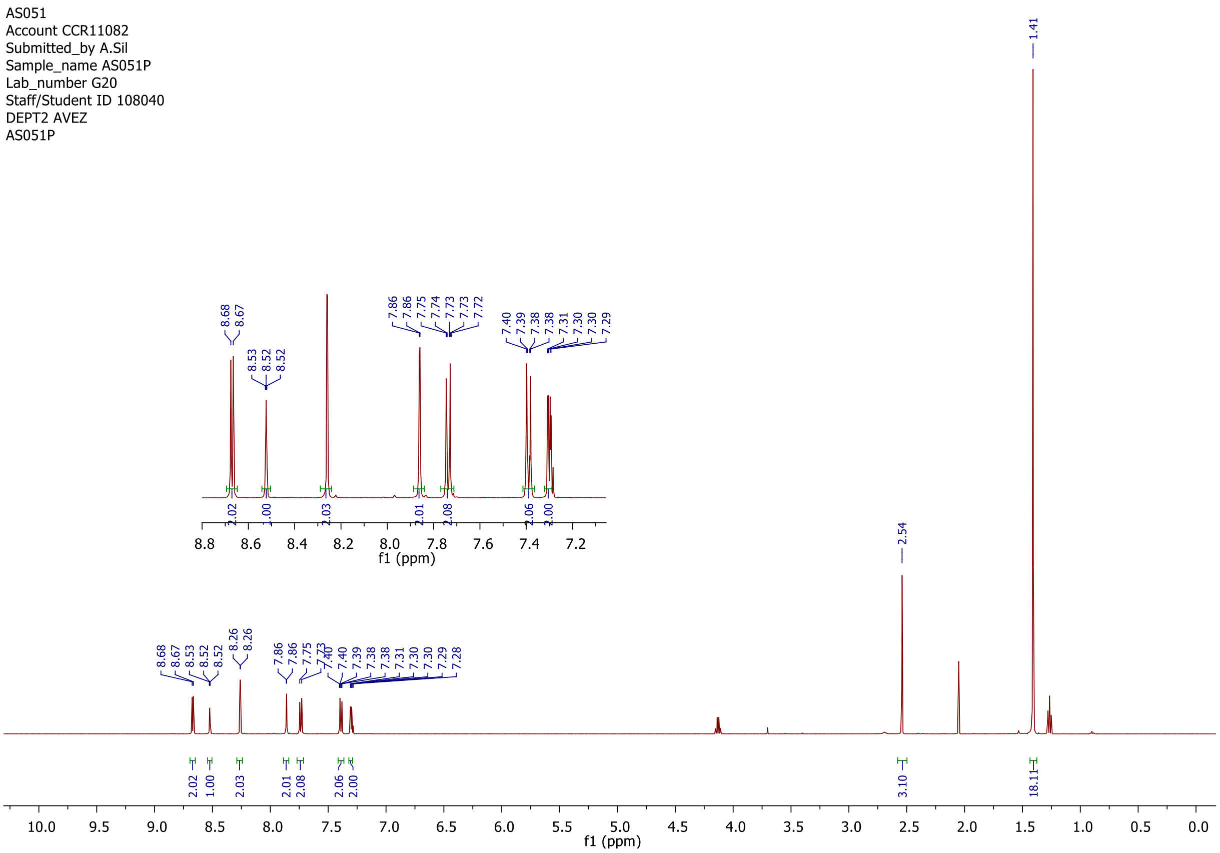

Figure S12: ^{1}H NMR (500 MHz, $CDCl_3$) of **L2**

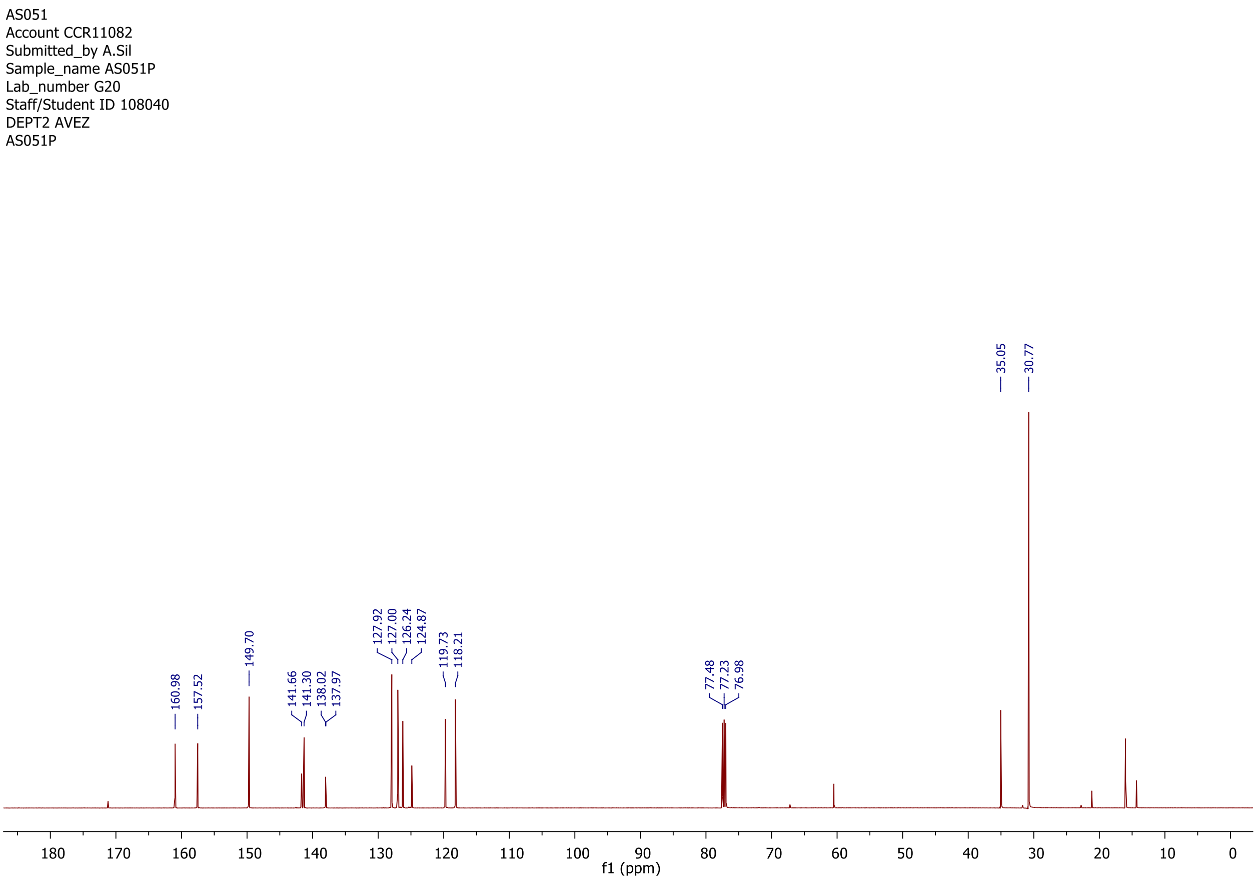

Figure S13: ^{13}C NMR (126 MHz, $CDCl_3$) of **L2**

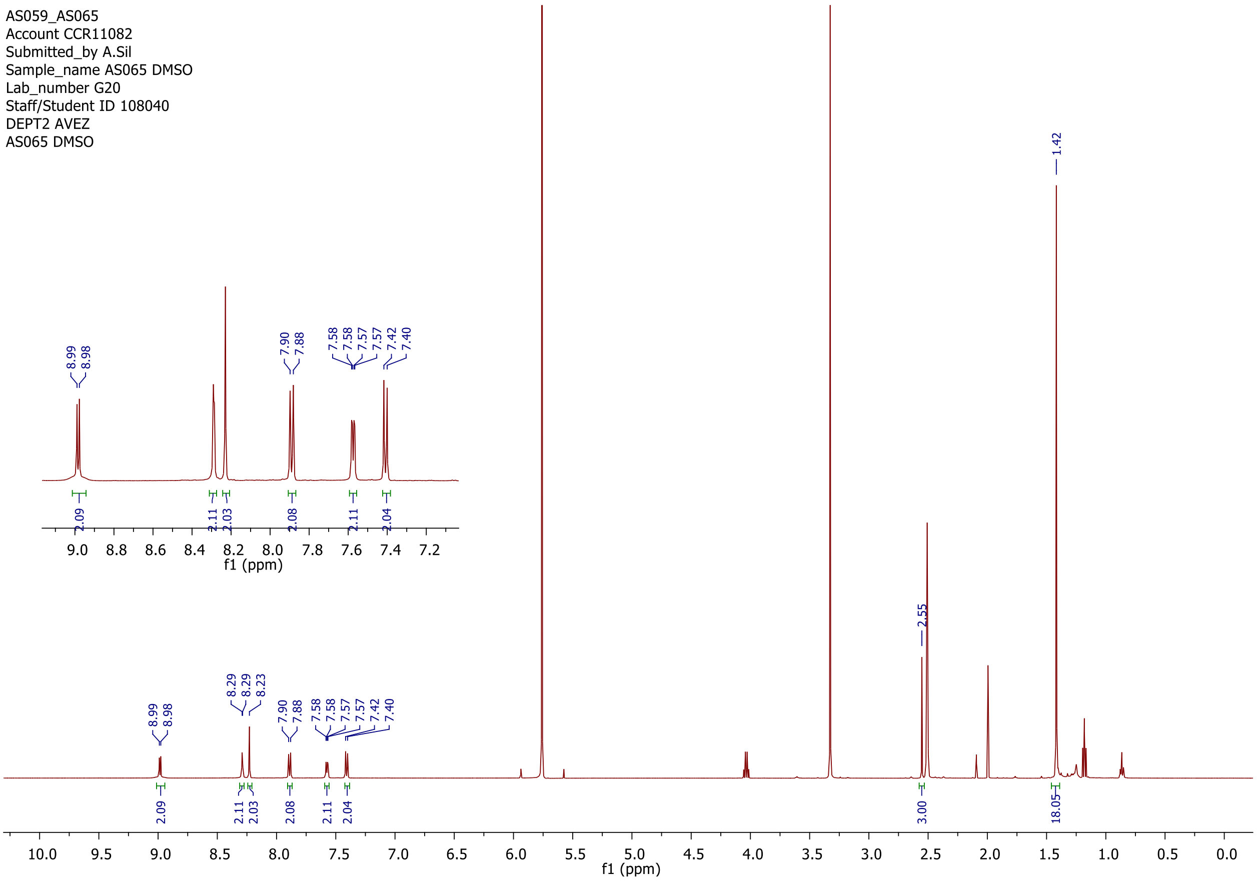

Figure S14: ^{1}H NMR (500 MHz, DMSO-d6) of **L2PtCl.** Unmarked peaks are residual solvents that could not be removed even after overnight drying *in vacuo* (*n*-hexane: 0.86, 1.25 ppm; DMSO: 2.50 ppm; CH_2Cl_2: 5.76; ethyl acetate: 1.17, 1.99, 4.03, acetone: 2.09 ppm).

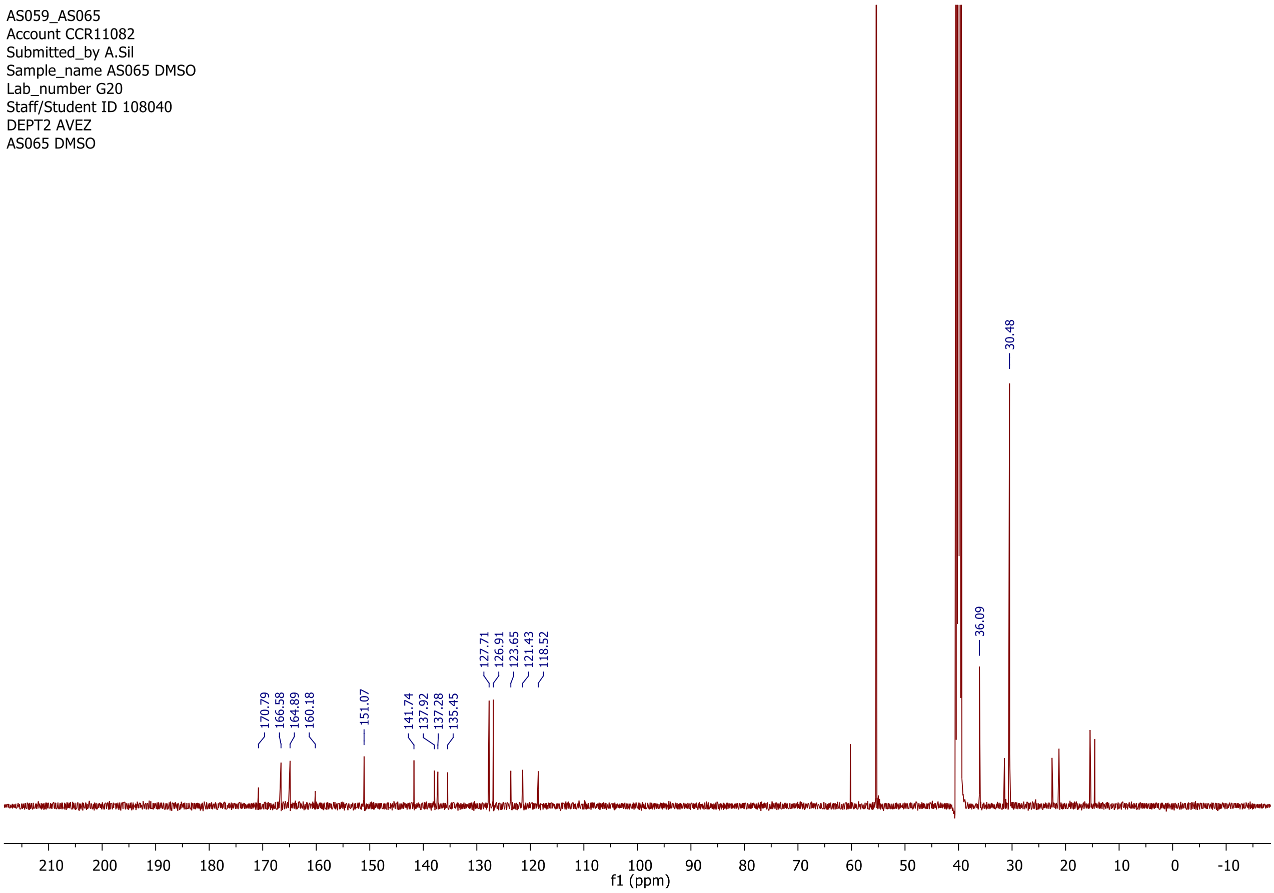

Figure S15: ^{13}C NMR (126 MHz, DMSO-d6) of **L2PtCl.** Unmarked peaks are residual solvents that could not be removed even after overnight drying *in vacuo* (*n*-hexane: 13.88, 22.05, 30.95 ppm; DMSO: 39.52 ppm; CH_2Cl_2: 54.84; diethyl ether: 15.12, 62.05 ppm).

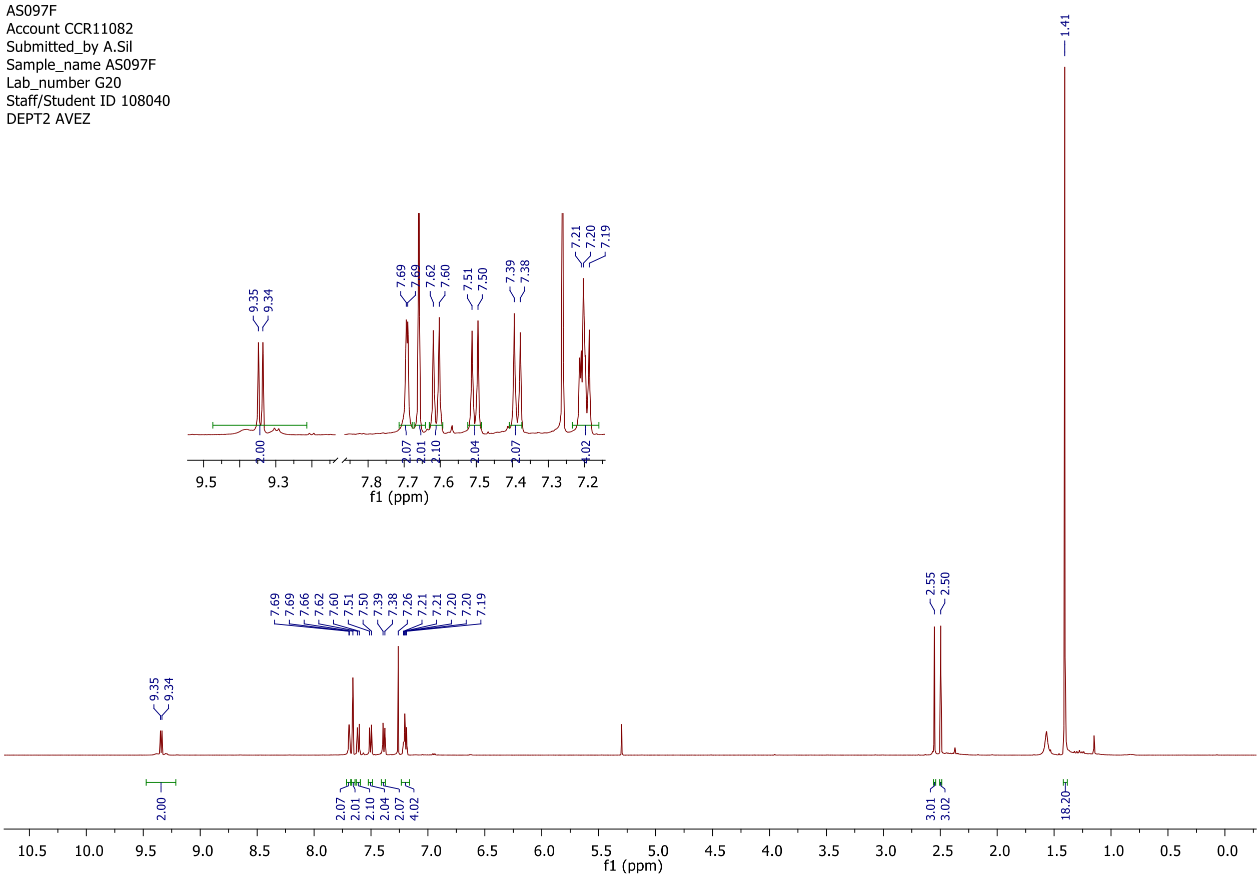

Figure S16: ^{1}H NMR (500 MHz, $CDCl_3$) of **L2PtT**

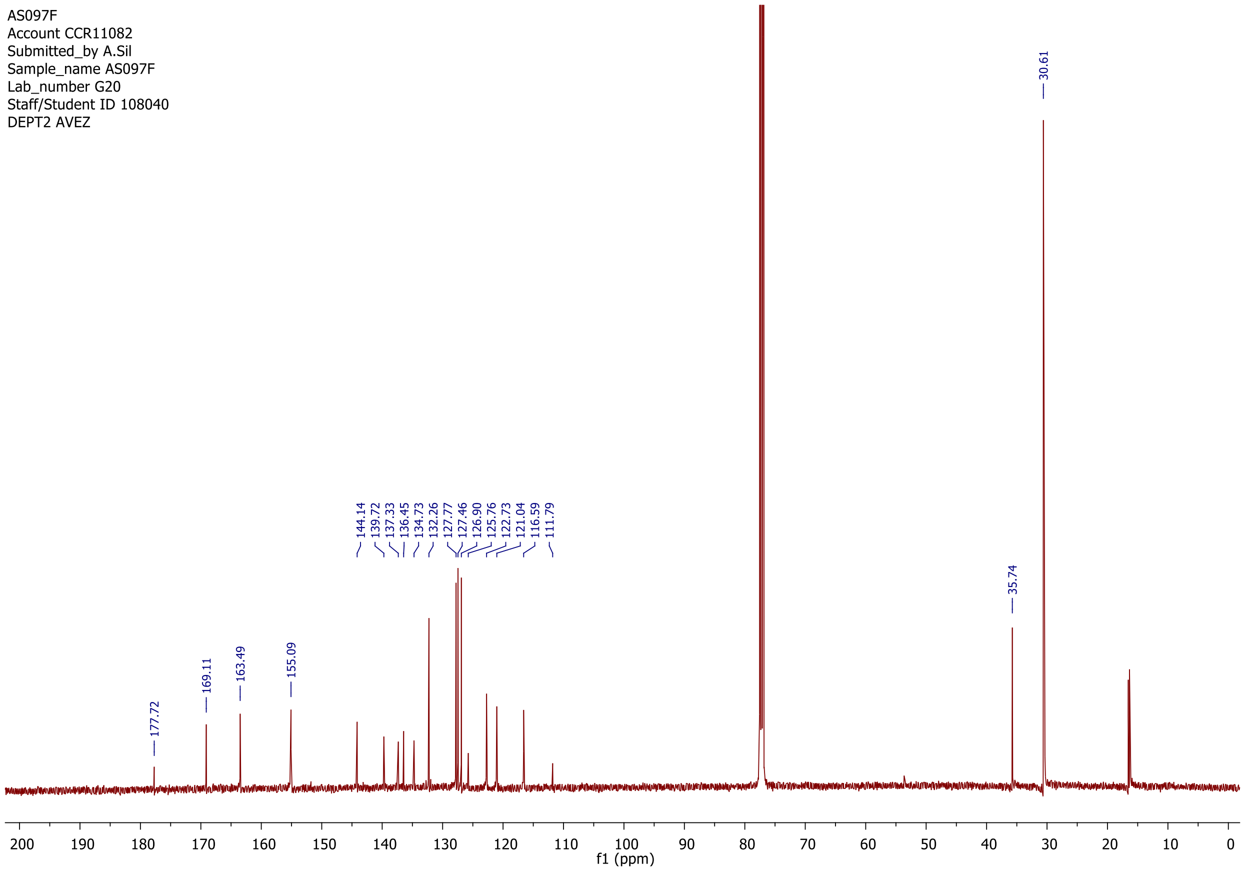

Figure S17: ^{13}C NMR (126 MHz, $CDCl_3$) of **L2PtT**

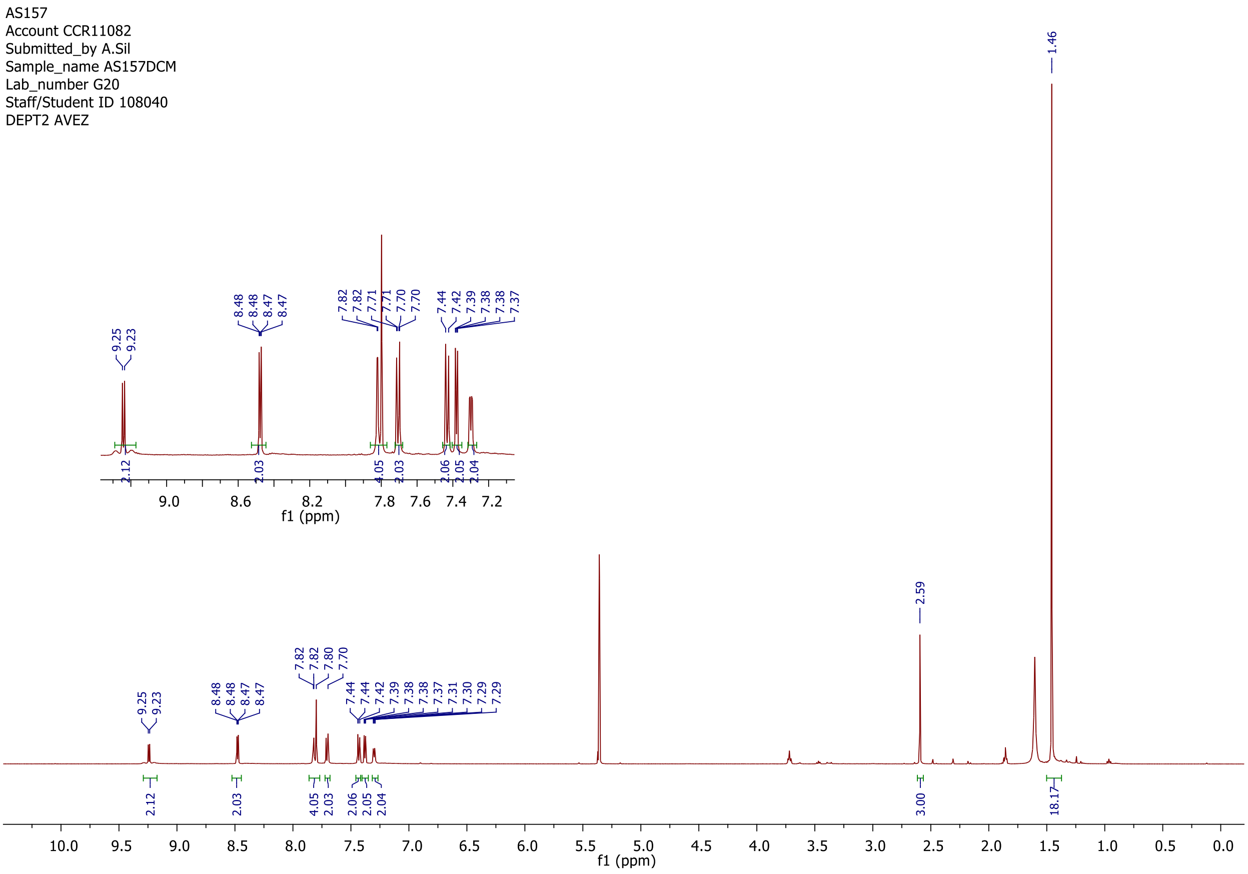

Figure S18: ^{1}H NMR (500 MHz, CD_2Cl_2) of **L2PtP**

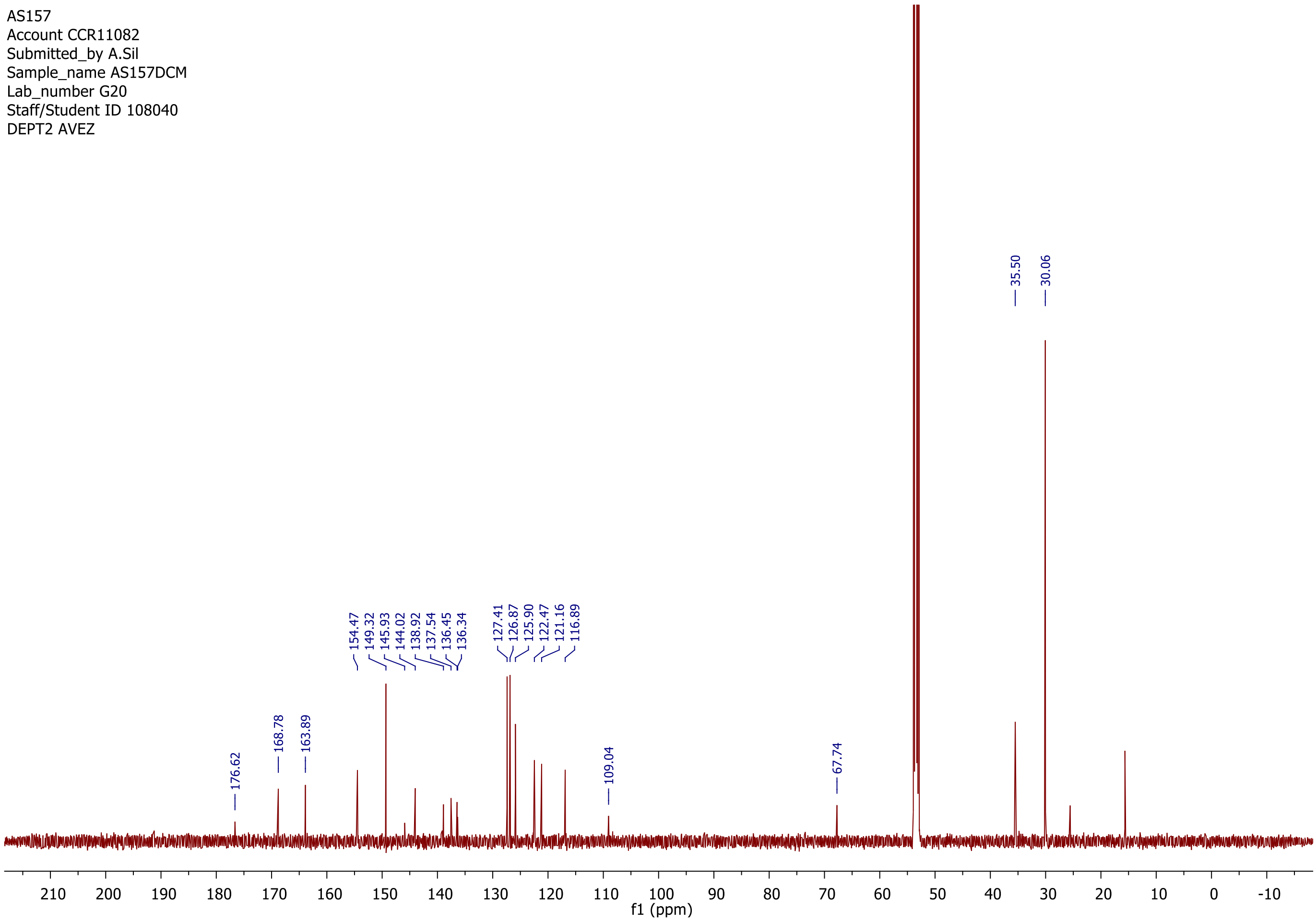

Figure S19: ^{13}C NMR (126 MHz, CD_2Cl_2) of **L2PtP**

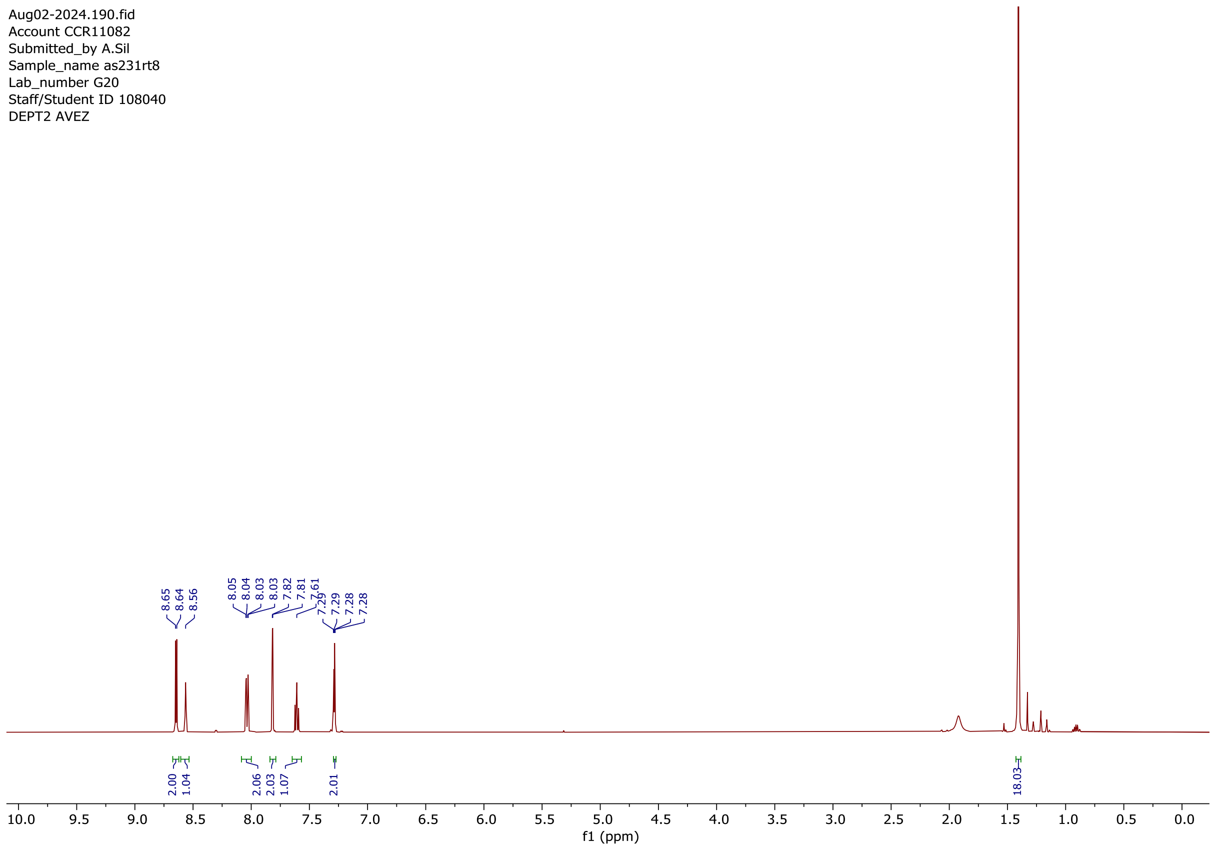

Figure S20: ^{1}H NMR (500 MHz, $CDCl_3$) of **L3**

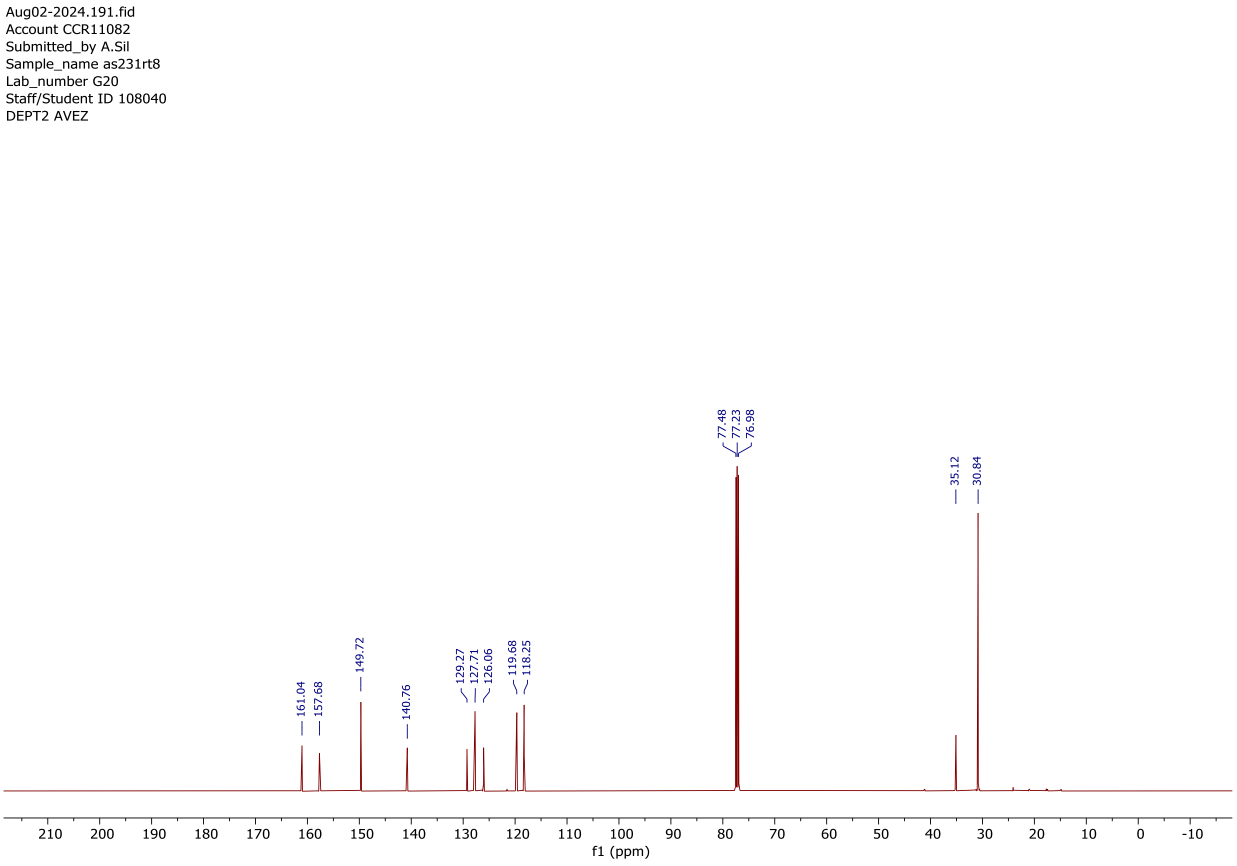

Figure S21: ^{13}C NMR (126 MHz, $CDCl_3$) of **L3**

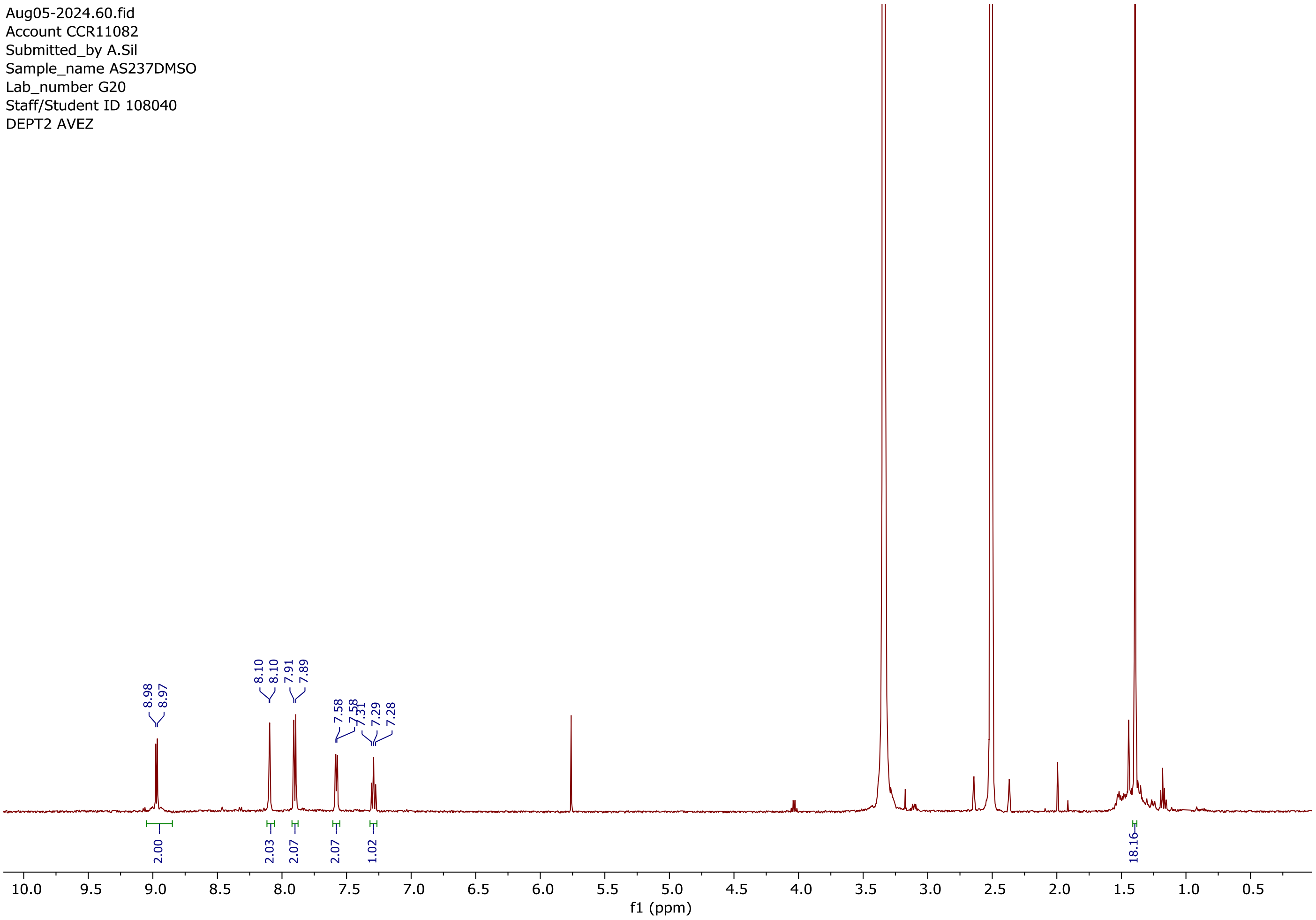

Figure S22: ^{1}H NMR (500 MHz, DMSO-d6) of **L3PtCl**

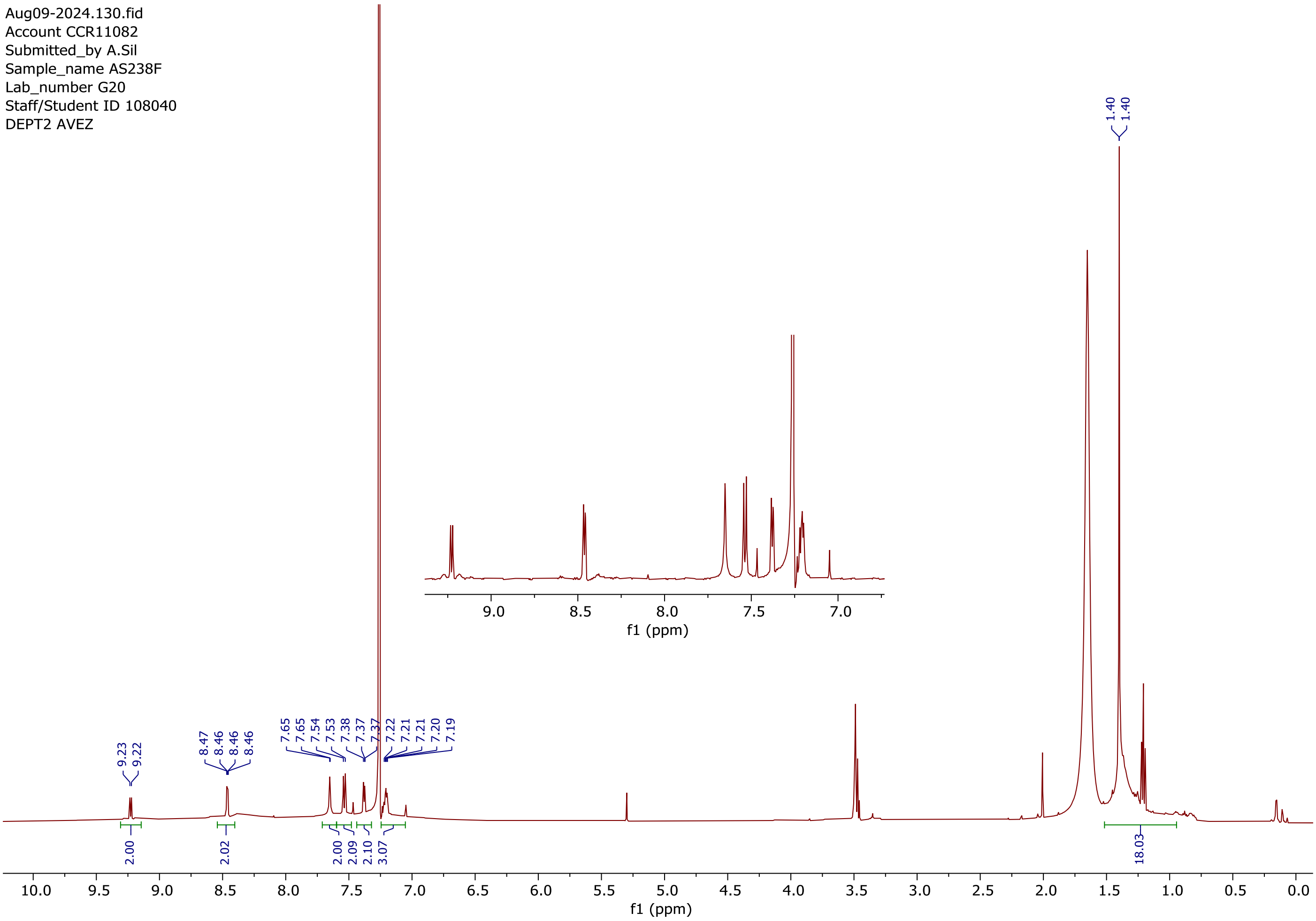

Figure S23: ^{1}H NMR (500 MHz, $CDCl_3$) of **L3PtP**

3. Details on the STMBJ technique and additional STMBJ Data

3.1 Technical details

We used a modified Keysight 5500 STM in our experiments. The STM is currently equipped with a Femto DLPCA-200 transimpedance amplifier (operating at $10^6\ V/A$ amplification). Voltages (substrate bias and piezoelectric transducer signal) are imposed by an arbitrary waveform generator (AWG; Keysight 33522B) and all signals are simultaneously acquired at 24-bit resolution by a National Instruments PXI System through a PXI-4464 board, at $20\ kSa/s$. The current signal is fed back into the STM controller to exploit the built-in PID feedback loop for tip approach and imaging. A set of resistors between the AWG and the substrate prevents overload of the amplifier by limiting the current through the system when $G \gg G_0$. The presence of a resistor ($200\ k\Omega$ in this work) causes a bias drop as the conductance of the junction is $> 0.1\ G_0$, allowing us to probe conductance over > 7 orders of magnitude with a single-channel preamplifier. A schematic of the instrument used is provided in Figure S24. Data acquisition and analysis are performed in LabVIEW, through bespoke VIs.

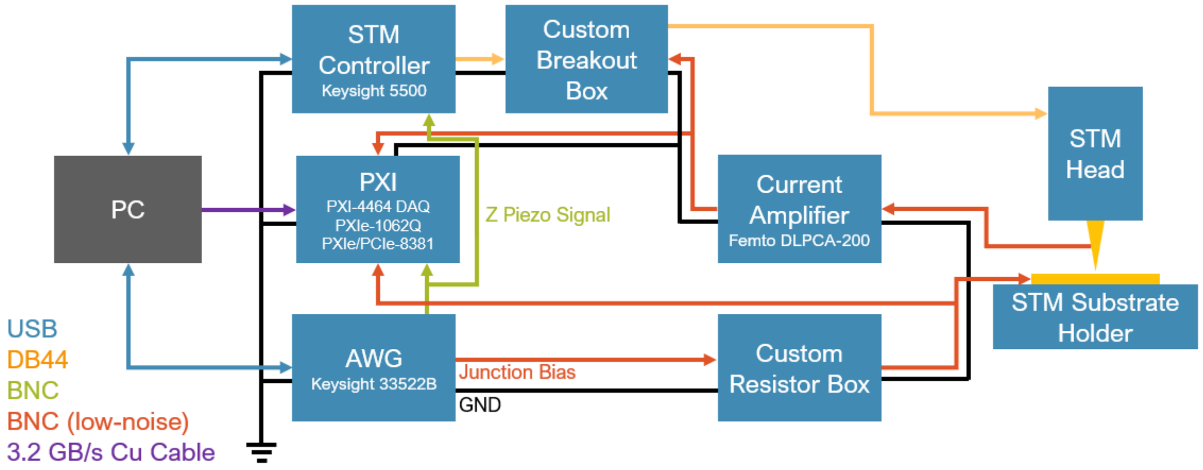

Figure S24: Schematic of the instrument used in this study.

All experiments in this study have been performed with Au tip cut from a spool of 99.998%+ Au wire (ThermoFisher Scientific PREMION), and substrates prepared by e-beam evaporation of 99.99+% Au (pellets, Advent Research Materials) on freshly cleaved muscovite mica (Agar Scientific). Substrate were briefly annealed with a butane torch before use. Mesitylene 98+% was purchased from TCI UK and used without further purification.

Further details are also provided in our previous publications.[3,4]

3.2 Data Analysis

STMBJ: During collection, each trace is checked to ensure that the conductance of the device is $G > 5G_0$ a the beginning of the piezo ramp (to ensure that a clean bulk microcontact has been fabricated), and that it decays at the noise level of the instrumentation ($10^{-5.2} - 10^{-6.5}$ depending on the bias applied and the transimpedance used) at the end of the ramp. STMBJ traces that do not fulfil these criteria are discarded and are not used to compile the histograms and data plot.

PM-STMBJ: In piezo-modulation experiments, data is instead selected using criteria we developed and described in detail in our previous publications where we used this technique.[5,6] In brief, modulation cycles (*e.g.* $8 \times 0.5\ nm$) are cut by calculating the second derivative of the piezo signal and gating the traces when a certain threshold is reached. Each modulation cycle is checked at its beginning and at end its to verify that the conductance of the trace is within the envelope of the corresponding peak in the conductance histogram, using $x_C \pm 2\sigma$ where x_C is the centre of the Gaussian distribution and σ is its standard deviation. When $x_C - 2\sigma$ extends beyond the noise level of our instrumentation, we used the value of the noise level as lower boundary. Only traces that satisfy these criteria are used to compile the piezo-modulation density maps. The process removes (i) traces where no junctions were formed and (ii) traces where the junction did not survive the full modulation cycle. Information on the obtained statistics is provided below, and all compounds returned a hitrate ($\frac{\#_{selected}}{\#_{acquired}}$) between 20% and 30%, consistent with our previous studies.[5,6]

Plateau Length Analysis: To extract the distributions of break-off distances from our datasets, we used an automated algorithm to isolate the portion of the trace with the plateau, by cutting between two conductance thresholds and trimming by 10 datapoints on each side (to remove noisy artifacts). The first threshold is $0.1\ G_0$, marking the beginning of the plateau, and the second is either the noise level of the instrument or the natural break in conductance between two contributions in the histogram (values used are reported in the caption of the break-off plots in the next section. To remove traces with no molecule assembled in the gap between the nanoelectrodes, the algorithm calculates the slope of the plateau and only selects those with slope < 8. Pure tunnelling has slope ~ 10. Histograms of the slope distribution show a natural break at this value, useful to separate traces bearing molecular signature from those arising from pure tunnelling.

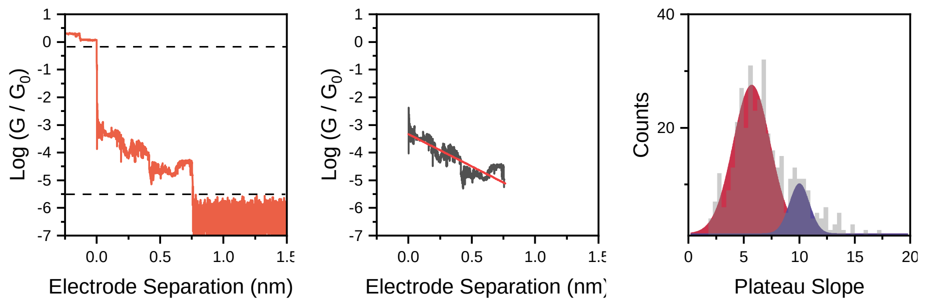

Figure S25: Plateau length analysis. (left) example STMBJ trace with example thresholds shown as dashed lines. (centre) resulting isolated plateau and its linear fitting. (right) example distribution of linear fitting slopes.

The length of the selected plateaux is then calculated and plotted as histograms. Fitting to a Gaussian distribution yields the most probable break-off distance and its standard deviation.

All LabVIEW virtual instruments to perform these analyses are provided under a Creative Commons license in the Liverpool Data Catalogue entry associated to this manuscript (see main paper for address and DOI).

3.3 Additional STMBJ Data

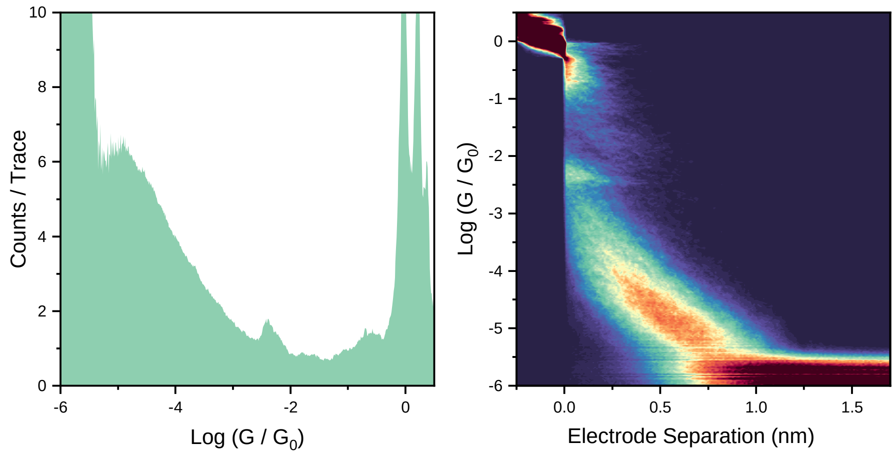

Figure S26: STMBJ conductance histogram and 2D density map for **L1**. 300 mV bias, 4279 traces used. 100 bins per conductance decade and 100 bins per nanometre. 0.1 mM in mesitylene.

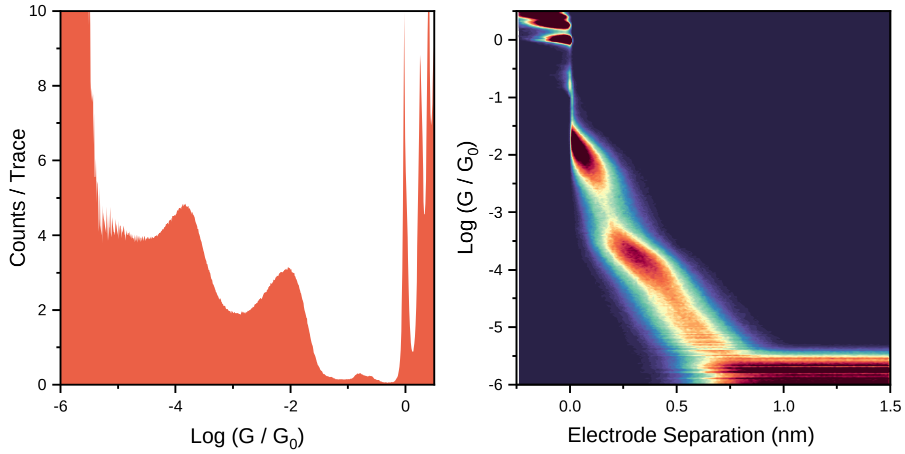

Figure S27: STMBJ conductance histogram and 2D density map for **L1PtCl**. 300 mV bias, 5878 traces used. 100 bins per conductance decade and 100 bins per nanometre. 0.1 mM in mesitylene.

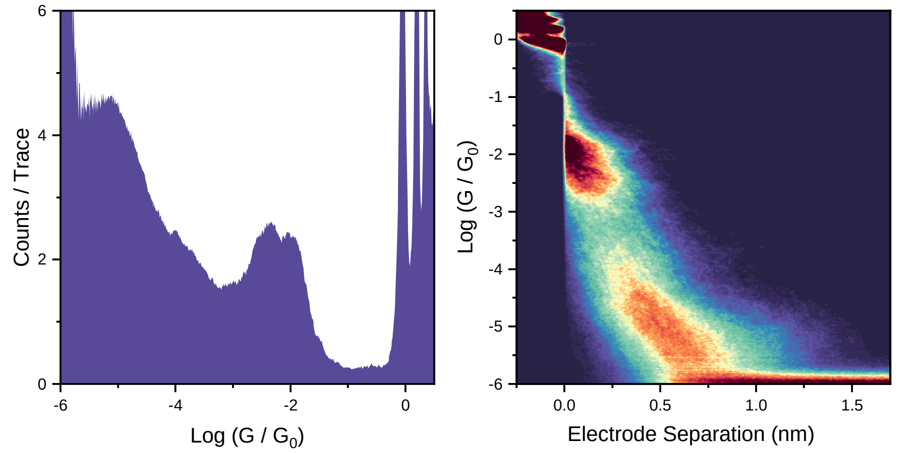

Figure S28: STMBJ conductance histogram and 2D density map for **L2PtT**. 600 mV bias, 5002 traces used. 100 bins per conductance decade and 100 bins per nanometre. 0.1 mM in mesitylene.

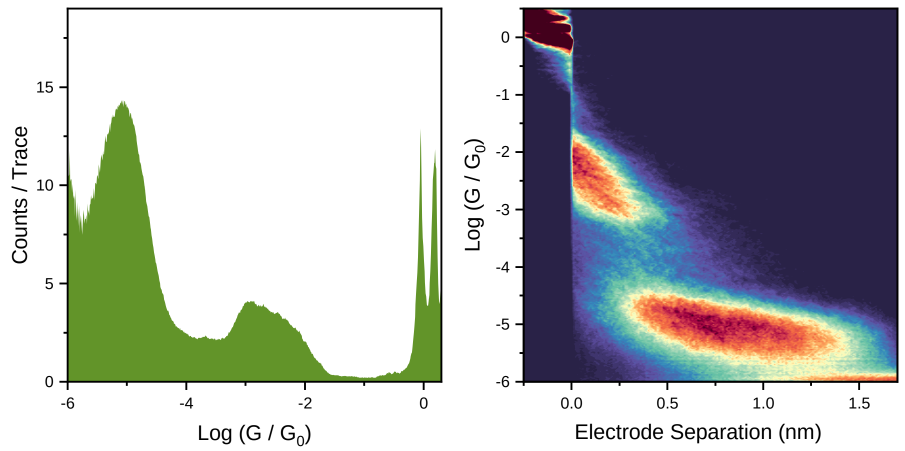

Figure S29: STMBJ conductance histogram and 2D density map for **L2PtP**. 600 mV bias, 5792 traces used. 100 bins per conductance decade and 100 bins per nanometre. 0.1 mM in mesitylene.

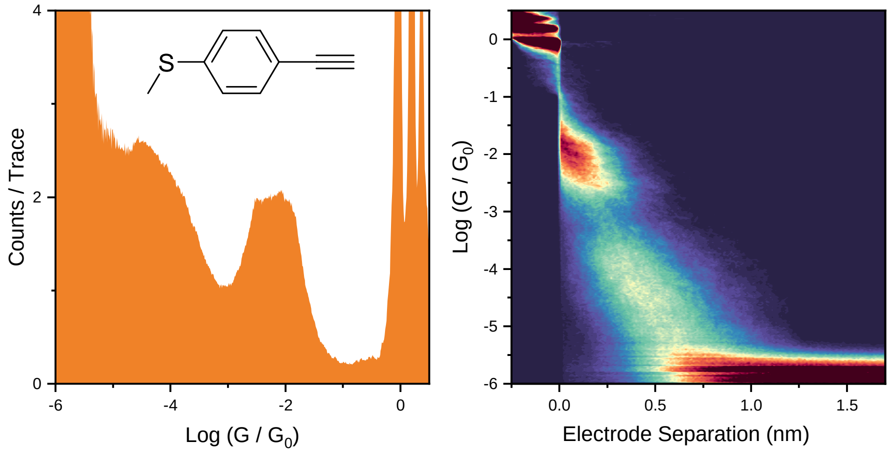

Figure S30: STMBJ conductance histogram and 2D density map for 4-ethynylthioanisole (structure inset). 300 mV bias, 6278 traces used. 100 bins per conductance decade and 100 bins per nanometre. 0.1 mM in mesitylene.

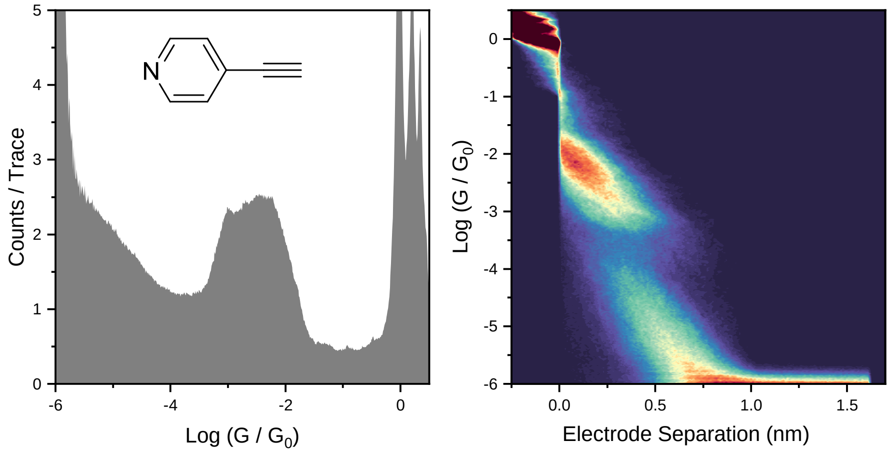

Figure S31: STMBJ conductance histogram and 2D density map for 4-ethynylpyridine (structure inset). 600 mV bias, 5795 traces used. 100 bins per conductance decade and 100 bins per nanometre. 0.1 mM in mesitylene.

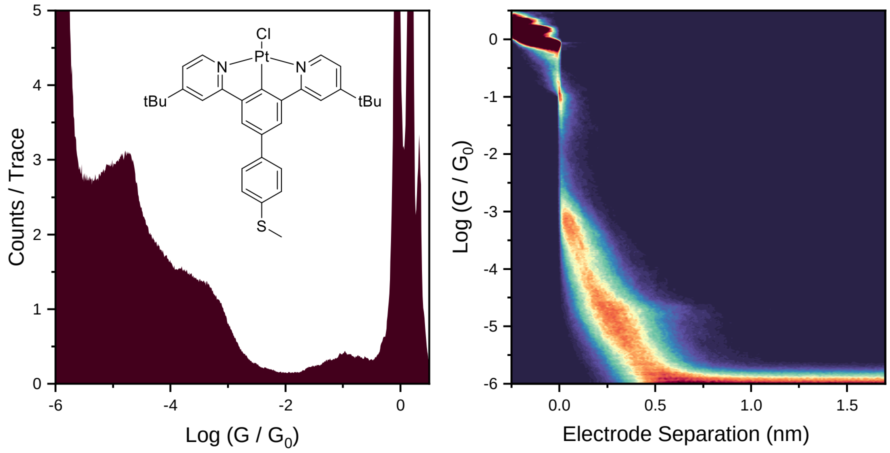

Figure S32: STMBJ conductance histogram and 2D density map for **L2PtCl** (structure inset). 600 mV bias. 3975 traces used. 100 bins per conductance decade and 100 bins per nanometre. 0.1 mM in mesitylene.

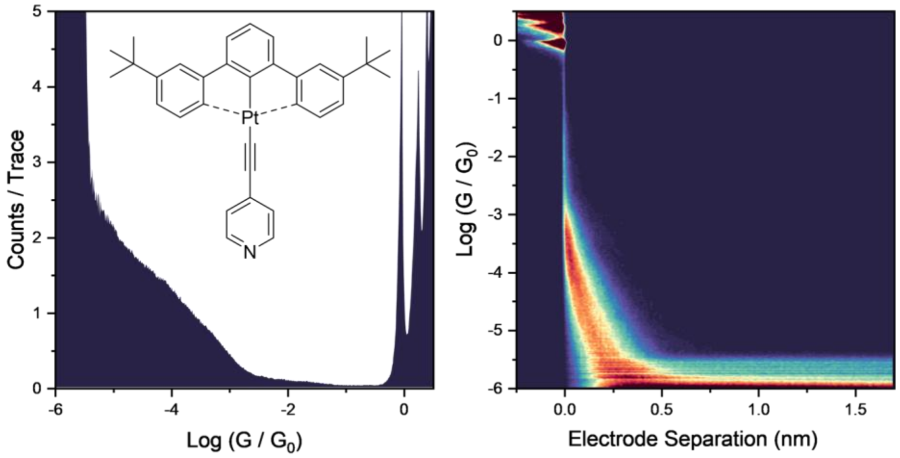

Figure S33: STMBJ conductance histogram and 2D density map for **L3PtP** (structure inset). 600 mV bias. 5325 traces used. 100 bins per conductance decade and 100 bins per nanometre. 10 μM in mesitylene.

3.4 Break-Off Analysis

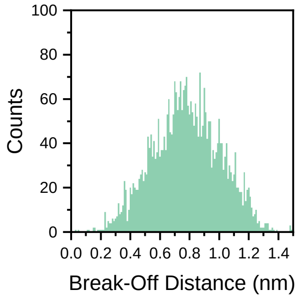

Figure S34: Break-off distance distribution for the STMBJ plateaux of **L1**. Plateau length was estimated by cutting the trace when G fell below the noise level of our instrumentation ($10^{-5.4}$ at $300\ mV$ bias). Break-off distribution is $0.77\ \pm 0.21\ nm$.

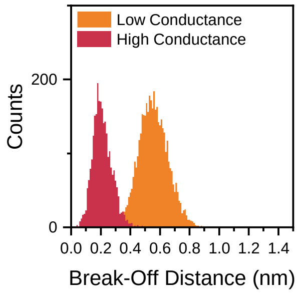

Figure S35: Break-off distance distribution for the high and low conductance plateaux of **L1PtCl**. High conductance plateaux were cut when $G \leq 10^{-2.9}\ G_0$. Low conductance plateaux were cut when G fell below the noise level of our instrumentation ($10^{-5.4}$ at $300\ mV$ bias). Break-off distributions are $0.20\ \pm 0.06\ nm$ and $0.58\ \pm 0.11\ nm$, respectively, for high and low conductance.

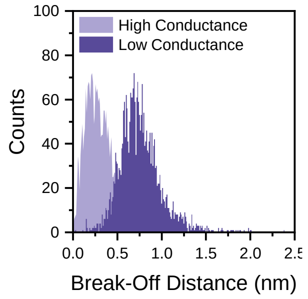

Figure S36: Break-off distance distribution for the high and low conductance plateaux of **L2PtT**. High conductance plateaux were cut when $G \leq 10^{-3.5}\ G_0$. Low conductance plateaux were cut when G fell below the noise level of our instrumentation ($10^{-5.9}$ at $600\ mV$ bias). Break-off distributions are $0.25\ \pm 0.15\ nm$ and $0.76\ \pm 0.17\ nm$, respectively, for high and low conductance.

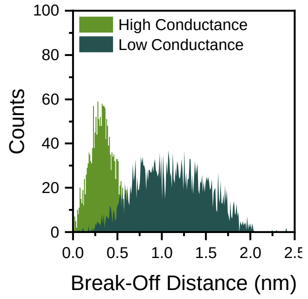

Figure S37: Break-off distance distribution for the high and low conductance plateaux of **L2PtP**. High conductance plateaux were cut when $G \leq 10^{-3.75}\ G_0$. Low conductance plateaux were cut when G fell below the noise level of our instrumentation ($10^{-5.9}$ at $600\ mV$ bias). Break-off distributions are $0.28\ \pm 0.15\ nm$ and $1.12\ \pm 0.48\ nm$, respectively, for high and low conductance.

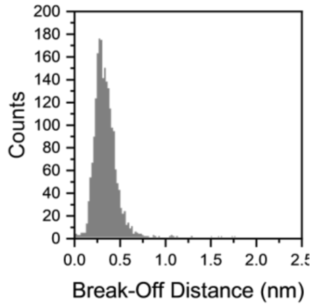

Figure S38: Break-off distance distribution for the STMBJ plateaux of **L2PtCl**. Plateaux were cut when G fell below the noise level of our instrumentation ($10^{-5.5}$ at $600\ mV$ bias). The break-off distribution is $0.30\ \pm 0.11\ nm$

3.5 Data Tables

Table S1: Comparison of experimental and theoretical junction lengths. (a) a snapback value of 0.5 nm was considered in this study. (b) Calculated by DFT, Q-CHEM 5.4.2, ωB97X-D/6-31G* level of theory. (c) S1 is the methyl sulfide on the acetylide side. (d) N is the pyridinyl nitrogen on the acetylide side.

**Compound**	**Experimental Breakoff (nm)**	**Junction length (including snapback)[a] (nm)**	**Theoretical Lengths[b] (nm)**
**L1**	0.77 ± 0.21	1.27 ± 0.21	1.26 (S-S)
**L1PtCl**	0.58 ± 0.11<br>0.20 ± 0.06	1.08 ± 0.10<br>0.70 ± 0.06	1.12 (S-S)<br>0.68 (S-Pt)
**L2PtT**	0.76 ± 0.17<br>0.25 ± 0.15	1.26 ± 0.17<br>0.75 ± 0.15	2.01 (S-S)<br>0.93 (S-Pt)[c]
**L2PtP**	1.12 ± 0.48<br>0.28 ± 0.15	1.62 ± 0.48<br>0.78 ± 0.15	1.83 (S-N)<br>0.75 (N-Pt)[d]
**L2PtCl**	0.30 ± 0.21	0.80 ± 0.11	1.09 (S-Pt)

Table S2: Statistical information on the piezo-modulation 2D density maps

**Compound**	**Modulation**	**# of traces acquired**	**# of traces selected**	**Hitrate**
**L1PtCl**	4 x 0.4 nm	11003	2820	25.6 %
**L1PtCl**	8 x 0.5 nm	13015	3626	27.8 %
**L2PtP**	4 x 1.0 nm	11093	2262	20.4 %

4. DFT Calculations

4.1. Methods

The geometries of the molecular structures studied in this paper were relaxed to a force tolerance of 10 meV °A^{-1} using the SIESTA[7] implementation of density functional theory (DFT), with a double-zeta polarized basis set (DZP) and the Generalized Gradient Approximation (GGA) functional with Perdew–Burke–Ernzerhof (PBE) parameterization. A real-space grid was defined with an equivalent energy cut-off of 150 Ry. To calculate the electronic properties of the junctions, from the converged DFT calculation, the Hamiltonian was combined with our quantum transport code, GOLLUM.[8] This yields the transmission coefficient T(*E*) for electrons of energy E via the relation T(*E*) = Trace $(\Gamma_R(E)G^R(E)\Gamma_L(E)G^{R+})$ where $\Gamma_{L,R}(E) = i(\Sigma_{L,R}(E) - \Sigma_{L,R}^{\dagger}(E))$ the level broadening due to the coupling between left L and right R electrodes and the central scattering region, $\Sigma_{L,R}(E)$ are the retarded self-energies associated with this coupling, and $G_R = (ES - H - \Sigma_L - \Sigma_R)^{-1}$ is the retarded Green's function, where H is the Hamiltonian and S is the overlap matrix obtained from SIESTA implementation of DFT.

4.2 Additional DFT Calculations

In addition to the energy diagram for **L1** and **L1PtCl** presented in the main text, the calculated conductance values are shown in Figure S39.

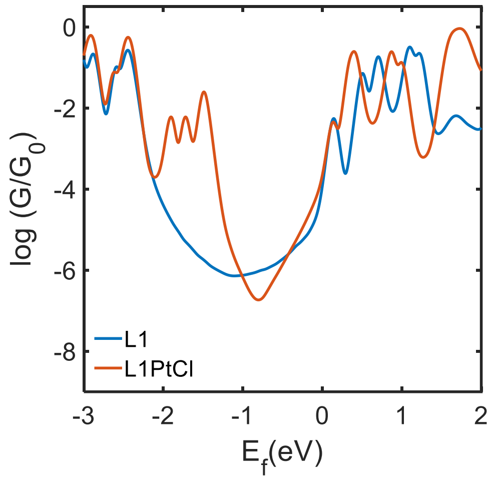

Figure S39: DFT electrical conductance curves for **L1** (blue) and **L1PtCl** (red).

We also computed $G(E)$ curves for **L2PtT**, and its comparison with **L2PtP** is shown in Figure S40. Our calculations are in good agreement with the experimental data, returning very similar values of conductance at the DFT-predicted Fermi energy, also accurate in their magnitude, both in the range of $10^{-4} - 10^{-5}\ G_0$.

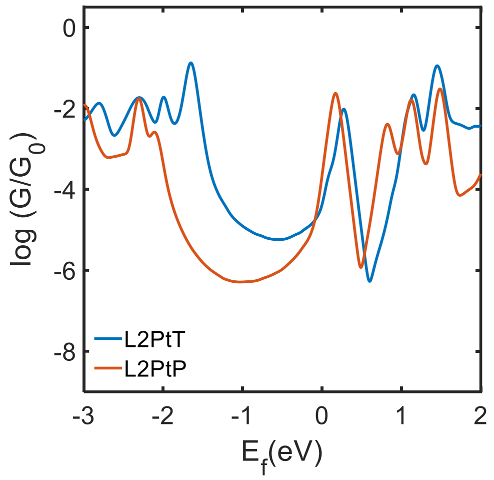

Figure S40: DFT electrical conductance curves for **L2PtT** (blue) and **L2PtP** (red)

We then proceeded to perform calculations on compressed **L2PtT** junctions, using the same geometry shown in the main paper for **L2PtP**. Again, our results show that a conductance increase is expected by Au-Pt(II) interaction, and this material should therefore show mechanoresistive phenomena similar to those observed for **L2PtP**. Unfortunately, these predictions were impossible to test experimentally due to the low-conductance feature of **L2PtT** being too close to the noise level of the instrumentation, causing a failure in our data analysis algorithm.

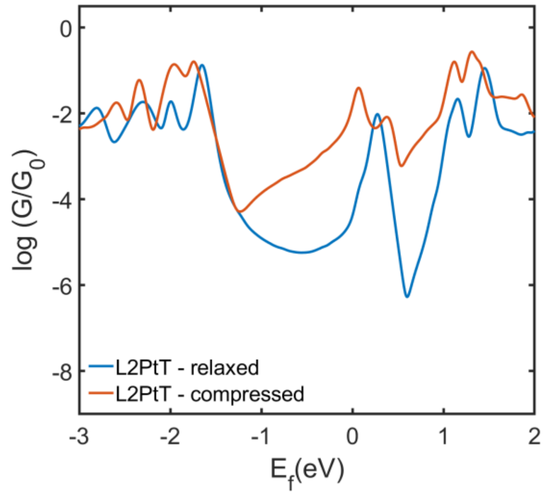

Figure S41: DFT electrical conductance curves for **L2PtT** in its relaxed (blue) and compressed (red) geometry.

Finally, to demonstrate the accuracy of our calculations, we show in the calculated room temperature conductance values across a range of energies around E_F. In this plot, the large dot is the value at E_F, while the small dots show the extent of variation in the range $\pm 0.1\ eV$. As can be seen, the trend of conductance is robust and in good agreement with the experimental results.

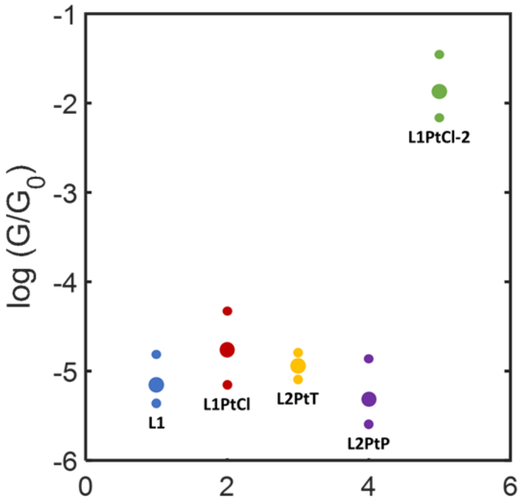

Figure S42: DFT-predicted room temperature conductance of **L1**, **L1PtCl**, **L2PtT**, **L2PtP** and **L1PtCl-2**, and the conductance variations for the Fermi energy range E_F-0.1 < E_F < E_F+0.1 eV. **L1PtCl-2** represents the compressed configuration, with the Au-Pt(II) electrode interface.

4.2 DFT orbitals

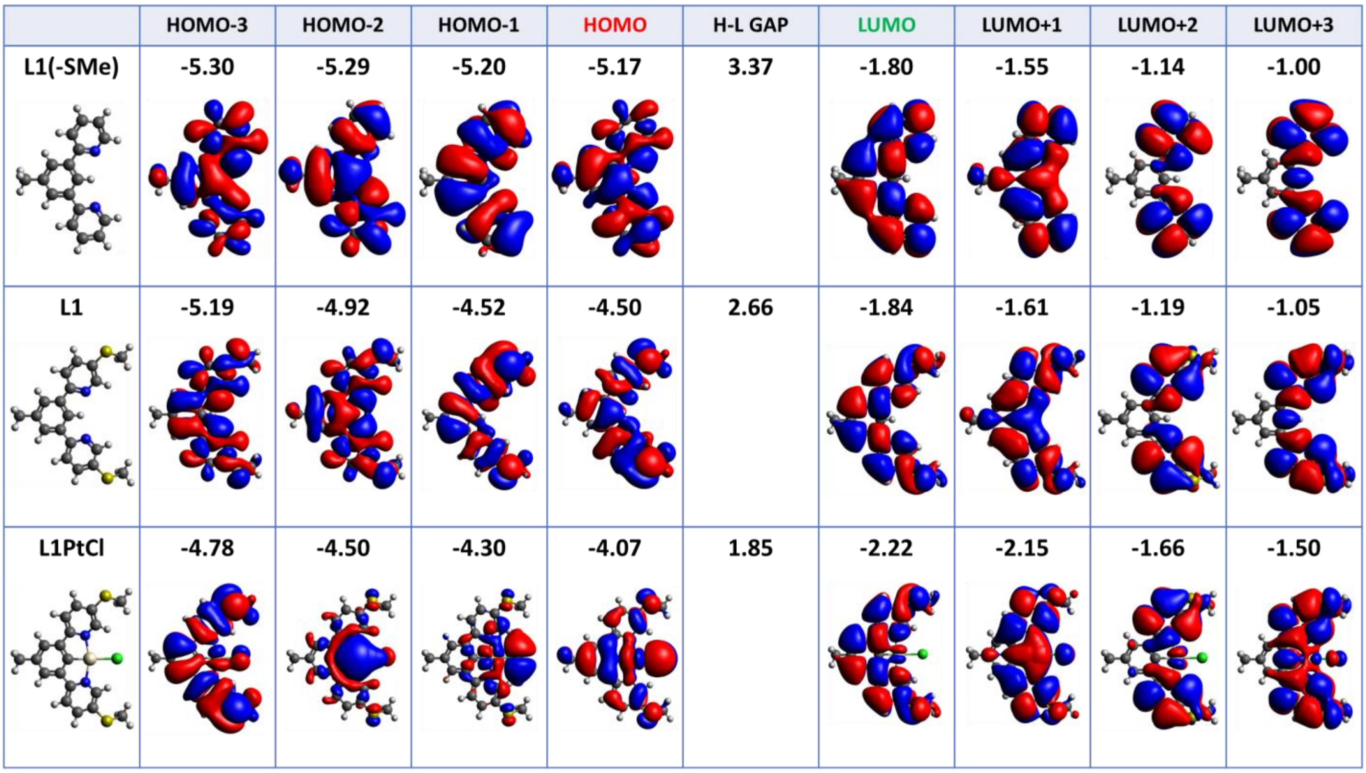

	HOMO-3	HOMO-2	HOMO-1	HOMO	H-L GAP	LUMO	LUMO+1	LUMO+2	LUMO+3
L1(-SMe)	-5.30	-5.29	-5.20	-5.17	3.37	-1.80	-1.55	-1.14	-1.00
L1	-5.19	-4.92	-4.52	-4.50	2.66	-1.84	-1.61	-1.19	-1.05
L1PtCl	-4.78	-4.50	-4.30	-4.07	1.85	-2.22	-2.15	-1.66	-1.50

Figure S43: DFT orbitals with corresponding energy values of **L1** without -SMe contacts, L1 and L1PtCl molecules.

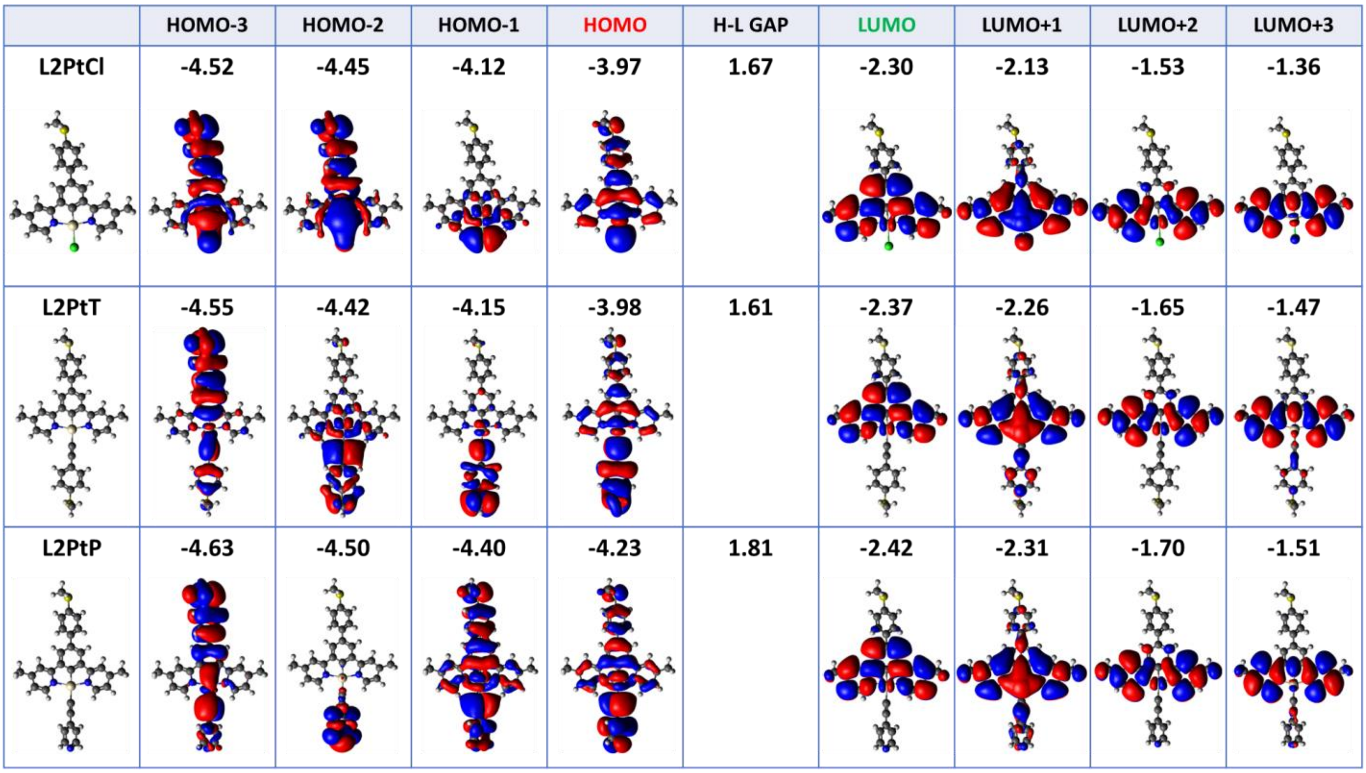

	HOMO-3	HOMO-2	HOMO-1	HOMO	H-L GAP	LUMO	LUMO+1	LUMO+2	LUMO+3
L2PtCl	-4.52	-4.45	-4.12	-3.97	1.67	-2.30	-2.13	-1.53	-1.36
L2PtT	-4.55	-4.42	-4.15	-3.98	1.61	-2.37	-2.26	-1.65	-1.47
L2PtP	-4.63	-4.50	-4.40	-4.23	1.81	-2.42	-2.31	-1.70	-1.51

Figure S44: DFT orbitals and corresponding energy values of **L2PtCl**, **L2PtT** and **L2PtP** molecules.

References

[1] M. Schmidt, D. Wassy, M. Hermann, M. T. González, N. Agräit, L. A. Zotti, B. Esser, E. Leary, *Chem. Commun.* **2021**, *57*, 745–748.

[2] E. Bosch, N. P. Bowling, J. Darko, *Cryst. Growth Des.* **2015**, *15*, 1634–1641.

[3] C. Wu, X. Qiao, C. M. Robertson, S. J. Higgins, C. Cai, R. J. Nichols, A. Vezzoli, *Angew. Chem. Int. Ed.* **2020**, *59*, 12029–12034.

[4] S. Naghibi, S. Sangtarash, V. J. Kumar, J.-Z. Wu, M. M. Judd, X. Qiao, E. Gorenskaia, S. J. Higgins, N. Cox, R. J. Nichols, H. Sadeghi, P. J. Low, A. Vezzoli, *Angew. Chem. Int. Ed.* **2022**, *61*, e202116985.

[5] N. Ferri, N. Algethami, A. Vezzoli, S. Sangtarash, M. McLaughlin, H. Sadeghi, C. J. Lambert, R. J. Nichols, S. J. Higgins, *Angew. Chem. Int. Ed.* **2019**, *58*, 16583–16589.

[6] C. Wu, D. Bates, S. Sangtarash, N. Ferri, A. Thomas, S. J. Higgins, C. M. Robertson, R. J. Nichols, H. Sadeghi, A. Vezzoli, *Nano Lett.* **2020**, *20*, 7980–7986.

[7] J. M. Soler, E. Artacho, J. D. Gale, A. García, J. Junquera, P. Ordejón, D. Sánchez-Portal, *J. Phys. Condens. Matter* **2002**, *14*, 2745–2779.

[8] J. Ferrer, C. J. Lambert, V. M. García-Suárez, D. Z. Manrique, D. Visontai, L. Oroszlany, R. Rodríguez-Ferradás, I. Grace, S. W. D. Bailey, K. Gillemot, H. Sadeghi, L. A. Algharagholy, *New J. Phys.* **2014**, *16*, 093029.
